# Ethnomycological knowledge among Kaqchikel, indigenous Maya people of Guatemalan Highlands

**DOI:** 10.1186/s13002-019-0310-7

**Published:** 2019-07-17

**Authors:** J. P. Mérida Ponce, M. A. Hernández Calderón, O. Comandini, A. C. Rinaldi, R. Flores Arzú

**Affiliations:** 1 Departamento de Microbiología, Facultad de CCQQ y Farmacia, Universidad de San Carlos de Guatemala, Ciudad Universitaria zona 12, 01012 Guatemala, Guatemala; 2 Department of Life Sciences and the Environment, University of Cagliari, Cittadella Universitaria, I-09042 Monserrato, CA Italy; 3 Department of Biomedical Sciences, Cittadella Universitaria, University of Cagliari, I-09042 Monserrato, CA Italy

**Keywords:** Ethnomycology, Mushroomers, Mushrooms, Secondary forest products, Maya culture, Mesoamerica

## Abstract

**Background:**

The Guatemalan Highlands is a region of great but so far poorly known mycological diversity. People living in this area have long used wild fungi as a source of food and income. However, our knowledge of the ethnomycological practices of the Mayan peoples of Guatemala is still rudimental, especially if compared with information reported for the neighboring region of Mexico. Among the main indigenous groups of the Maya people inhabiting the highlands of Central Guatemala, stand the Kaqchikel, accounting for nearly 8% of the entire Guatemalan population. The main aim of this study was to record the traditional knowledge and use of edible wild mushrooms by inhabitants of the municipality of San Juan Sacatepéquez that lies at the heart of the Kaqchikel area in the central highlands of Guatemala, also describing the relevant selling practices and dynamics. A secondary aim was to compare the diversity and composition of the mushroom assemblage offered at the market with the macrofungal diversity of woods in the area.

**Methodology:**

This study is the result of 4 years of ethnomycological research, conducted through continuous visits to the municipal market and focused interviews with collectors and vendors. Field sampling in pine-oak forested areas surrounding San Juan Sacatepéquez, from where the mushrooms sold at the market are foraged, were also conducted, in the presence of local collectors.

**Results:**

The results show a significant richness of species sold in the market, a network of commerce of purchase, sale, and resale of several species, with relatively stable prices, and knowledge about edible and inedible species that is transmitted mainly within the family nucleus. The business of selling mushrooms in the market is an exclusive activity of women, who are supplied by collectors or by other vendors. Fungi are sold and bought only as food, while no consumption of hallucinogenic mushrooms or medicinal mushrooms was recorded. Several species of *Amanita*, *Cantharellus*, *Boletus*, *Lactarius*, and *Russula* were those most commercialized in the 4 years of the study, but we also spotted fungi never reported before as consumed in the country, including *Gastropila* aff. *fumosa* (= *Calvatia fumosa*) and several species of *Cortinarius*. Field sampling in nearby pine-oak forests confirmed an elevated local macrofungal diversity.

**Conclusion:**

Our study unveiled the contemporary wealth of Kaqchikel culture for what concerns mushrooms, demonstrating that mushrooms continue to be culturally and economically important for these communities despite the erosion of traditional knowledge. Our results also confirmed the need to investigate in greater detail the Guatemalan mycodiversity that is vast and poorly known.

**Electronic supplementary material:**

The online version of this article (10.1186/s13002-019-0310-7) contains supplementary material, which is available to authorized users.

## Introduction

Thanks to its highly variable territory, Guatemala is one of the richest biodiversity hotspots in the world [1]. A vast array of ecosystems occurs from sea level up to more than 4000 m above sea level, including tropical and sub-tropical rain forests, wetlands, dry forests, scrublands, cloud forests, and pine-fir forests. This great biodiversity is matched by the unique ethnic and cultural composition of the country. The most populous country in Central America (about 15.5 million in 2017), Guatemala is home to several groups of indigenous people, mostly of Mayan ancestry (the 2003 Law of National Languages officially recognized 23 indigenous languages, including 21 of Maya origin, Xinka, and Garifuna); together, these groups account for some 40% of the overall population. Indigenous people often live in the most naturally valuable areas of Guatemala, that are also the most vulnerable ones, facing serious threats due to habitat loss, deforestation, over-exploitation of natural resources, and environmental contamination. Indigenous communities are thus both the custodians of Guatemalan biodiversity and those most directly affected by its demise.

The Kaqchikel are one of the main indigenous groups of the Maya people inhabiting the highlands of Central Guatemala (departments of Chimaltenango, Quiché, Guatemala, Sololá, Escuintla, and Sacatepéquez). They account for 8% of the Guatemalan population; some 400,000 Kaqchikel speak their native language, one of the four main Mayan languages (the other three being K’iche, Mam, and Q’eqchi’), which is actually divided in seven main dialects [2]. The economy of the Kaqchikel region is largely based on agriculture, basically centered around corn and beans; wood (pine and oak) is still the main fuel in homes for cooking and heating. Tourism is an increasingly important source of income, thanks to the beauty of landscape, archeological sites, baroque-colonial churches, syncretic traditions, and the rich-colored local dresses and markets. Economic pressure and the prolonged civil war (1960–1996) have forced many Kaqchikel to migrate, both towards urban centers and international destinations. Departure often results in migrants loosening their ties with the original communities, abandoning the Kaqchikel culture, traditional knowledge, and language [3].

Ethnomycology is a relatively new area of research that focuses on the study of the interrelations between human societies and fungi. The book published in 1957 by R. Gordon Wasson and his wife Valentina Pavlovna, *Mushrooms*, *Russia and History*, can be safely considered as the starting point of ethnomycology as a field of study [4]. The subjects of ethnomycology include cultural, ceremonial, and medicinal uses of mushrooms, besides their consumption as food [5, 6]. As such, ethnomycological surveys can help us to understand how traditional societies used to exploit biodiversity in their territories while preserving it, opening a window on ‘the world until yesterday’ [7]. The mycophilic, or even ‘mycolatry’, attitude of Mesoamericans is renowned, and dates back well to pre-Columbian times, as shown by mushroom stones common from Preclassic to Late Classic/Postclassic periods (1000 B.C.–1000 A.D., although not continuously, and with great variations in style) as well as by mushroom representations in the few surviving codices [8–11]. Most studies in the area have dealt with mushroom consumption and additional uses by ethnic groups in Mexico [12–14], while only limited attention has been devoted so far to ascertaining the ethnomycological knowledge of contemporaneous Guatemalan indigenous people (see [15] and references therein). In order to close this gap, we conducted a detailed investigation on the traditional mycological knowledge associated with the diversity of fungi, their use, trade, and beliefs of indigenous people in the municipality of San Juan Sacatepéquez (about 30 km from Guatemala City), at the heart of the Kaqchikel territory.

The main aims of the present work were (1) to record the knowledge and use of wild mushrooms as food by inhabitants of San Juan Sacatepéquez, both of Kaqchikel origin and ladinos; (2) to enquire the practices and dynamics of the wild mushroom selling process, including prices and purchase preferences; (3) to investigate about traditional beliefs surrounding mushrooms, and their eventual use for reasons other than food (e.g., medicinal); and (4) to ascertain the processes of intergenerational transmission of knowledge about wild mushrooms. Given the importance of San Juan Sacatepéquez as a market for edible mushrooms, supplied by foraging in the surrounding pine-oak forests, we expected to obtain a good amount of information about the aforementioned issues through direct contact and semi-structured interviews with both local mushroom vendors and collectors. To complement observations about edible mushrooms sold at the local market and to gather clues on the sustainability of the collection practices, we further aimed at conducting a preliminary survey of the macrofungal species present in pine-oak forests of the municipality.

## Materials and methods

### Study area

**Fig. 1:**
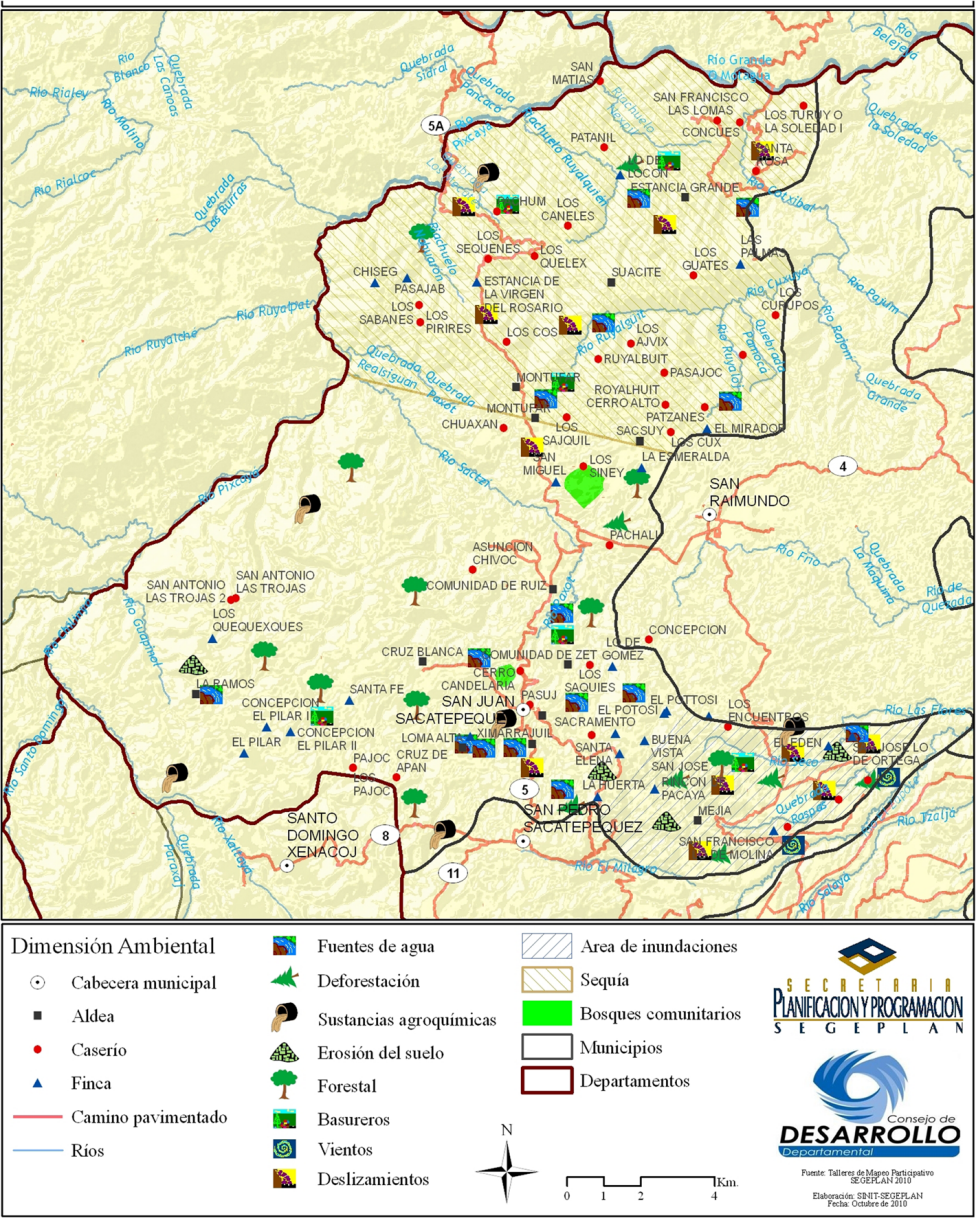
Map of San Juan Sacatepéquez municipality. From  http://sistemas.segeplan.gob.gt/sideplanw/SDPPGDM$PRINCIPAL.VISUALIZAR?pID=AMBIENTAL_IMG_110. Reproduced with permission

San Juan Sacatepéquez is located in the northeastern part of the department of Guatemala, in the northern part of the Panchoy Valley, 32 km from the capital, and has a territorial extension of 242 km^2^. It borders on the north with the municipality of Granados (department of Baja Verapaz), on the east with San Raymundo and San Pedro Sacatepéquez (department of Guatemala), to the south with Mixco and San Pedro Sacatepéquez, and to the west with El Tejar and San Martín Jilotepeque (department of Chimaltenango) and with Santo Domingo Xenacoj (department of Sacatepéquez) (Fig. 1).

The Municipality of San Juan Sacatepéquez consists of 20 villages (*aldeas*), 59 hamlets (*caseríos*), and several suburbs, with a total population of about 317,000 inhabitants [16]. Some 34% of the people in the municipality speak Kaqchikel as their mother language, but in the main urban center Spanish is also widely used. With its 1845 m of elevation, San Juan Sacatepéquez is a place of cold climate and of quite mountainous and broken terrain [17]. The local climate has varied significantly in the last decades due to deforestation and land use change. According to data from the Guatemalan Ministry of Agriculture, Livestock and Food (MAGA, http://web.maga.gob.gt/), three main ecosystems make the most of this territory: humid sub-tropical forest (temperate) with approximately 50% coverage, humid montane forest (sub-tropical) with 30%, and sub-tropical dry forest with 20%.

San Juan Sacatepéquez owes its name in honor of its patron saint, San Juan Bautista, whose patronal feast is on June 24. The word Sacatepéquez is composed of two voices of the old Pipil language, “*sakat*” grass and “*tepek*” hill, because during the conquest of Guatemala, Pedro de Alvarado brought indigenous guides from Mexico (Nahuatl and Pipiles) who named that place as “hill of grass” for its appearance and vegetal cover [18]. Regarding its pre-Hispanic origin, it is known that this was one of the main settlements that formed the Kaqchikel kingdom (eastern Kaqchikel group). The colonial city was founded on July 2, 1568, in the region of Yampuc, by Fray Benito de Villacañas [18], just a few years after the Conquest of Guatemala. Currently, the economy of the area is centered on the production of fruits and vegetables as well as flowers. Indeed, floriculture is the industry that represents the greatest economic wealth of the municipality. Other activities in order of importance are the manufacture of charcoal (exclusive of men), brick making, pottery, as well as typical fabrics (the local daily and the ceremonial *huipil*—women blouses—are among the most beautiful in the whole country) and the manufacture of wooden furniture.

### Field work

The ethnomycological survey was carried out from May to December 2012 and May to November 2015, through an oral interview to people who collect and/or sell mushrooms at the central market of San Juan Sacatepéquez. People were asked specifically about their knowledge of mushrooms, the ways this knowledge was acquired and transmitted, and the commerce of mushrooms in the San Juan Sacatepéquez area. The market of San Juan (as it is shortly named) is the reference market for a vast area in and around the main center of the municipality; it is specialized in the flower trade, but a range of other products can be found, including vegetables, fruits, grains, prepared food, household utensils, electronic items, and handicrafts.

We interviewed 14 mushroom vendors regularly settled in the plaza (all women, no male mushroom sellers exist here); some of them were also collectors. During periods of particular mushroom abundance, more women came to the plaza to sell them, especially *Cantharellus lateritius*, and we took the chance to interview some of them. We also asked 28 men workers at the Cementos Progreso plant in Chivoc village—which lies just beside the area of the cement plant and is one of the main places of gathering and home to a number of sellers/collectors—to fill a form with the same questions asked to vendors (see above). It should be noted that the survey of salespeople was done individually, one by one, recording their responses with their consent, while the workers of the plant only responded in writing to the provided document. The latter strategy was pursued in order to allow them to discuss questions at home, with their families, thus providing access to mycological knowledge shared by a larger community. Two local Mayan spiritual guides were also interviewed. We employed the participant observation method and applied semi-structured interviews with all sellers of wild mushrooms [19]. With the help of women volunteers and facilitators of the Carlos F. Novella Foundation, which has ongoing projects for the care of women in San Juan Sacatepéquez, and with the same mushroom sellers, we inquired about the ways of purchase and sale of mushrooms, prices, season of collection, traditional forms of preparation, use (medicinal, hallucinogenic, or some other traditional uses besides food, if any), and names of fungi in Kaqchikel and Spanish. A written comparison of the names in Kaqchikel was also made since there are dialectal varieties in Kaqchikel speech groups and also in Spanish. All oral interviews were recorded in digital format and archived.

The San Juan Sacatepéquez market was visited weekly during the survey period, 2012 and 2015, and occasionally in 2016–2017. All mushrooms offered for sale were bought through direct purchase from vendors who were present during the visit days. In addition, mushrooms were also collected in pine-oak forests in the area, with the help of five resident collectors and three local workers of the cement plant who have a deep knowledge of edible mushrooms in the zone; this was essential in order to describe the gathering process, applying the participant observation technique. Also, this permitted to identify other species of edible mushrooms that are collected and consumed in the area but not sold in the municipal market. All specimens, both from the market and collected in nature, were photographed and morphologically described. Identification was performed using field guides of mushrooms from North America [20–22] and specific studies previously conducted in Guatemala [15, 23, 24]. Samples were dried and admitted to the Rubén Mayorga Peralta mycoherbarium (MICG), Microbiology Department, Facultad de CCQQ y Farmacia, Universidad de San Carlos de Guatemala. Some species bought at the market were cooked following traditional methods as indicated by vendors/collectors and eaten by members of the research team [25].

## Results and discussion

### Edible mushrooms and local macrofungal diversity

The ethnomycological study carried out in the municipality of San Juan Sacatepéquez permitted to obtain important information about fungal diversity, the origin and forms of sale of edible mushrooms, prices and seasonality, the names in the Kaqchikel language and Spanish, the forms of consumption, and knowledge related to ecology and local 'cosmovision'. San Juan Sacatepéquez was selected due to its proximity to the capital city of the country, its large Kaqchikel population, its well-known market of edible mushrooms, and also because in the municipality, the traditional use of land is changing, with a noticeable reduction of the native pine-oak forests that once covered most of that mountainous territory. Regarding the origin of the mushrooms sold in the market of San Juan Sacatepéquez, it was determined that the majority are collected in the locations listed in Table 1.

**Table 1:** Main villages where edible mushrooms are collected in the Municipality of San Juan Sacatepéquez and sold in the market

Origin	
Aldea (village)	Caserío (hamlet)
Sajcavillá	
Loma Alta	
La Ramos	
Camino a San Pedro Sacatepéquez	
Cruz Blanca	Caserío San Antonio Las Trojes
	Finca Los Quequesques
Comunidad de Ruiz	Caserío Asunción Chivoc
Comunidad Zet	Caserío Cruz Verde
Finca El Pilar	Caserío Concepción El Pilar I
	Comunidad El Pilar II

Source: information obtained through interviews conducted in the frame of this study, during 2012 and 2015

**Fig. 2:**
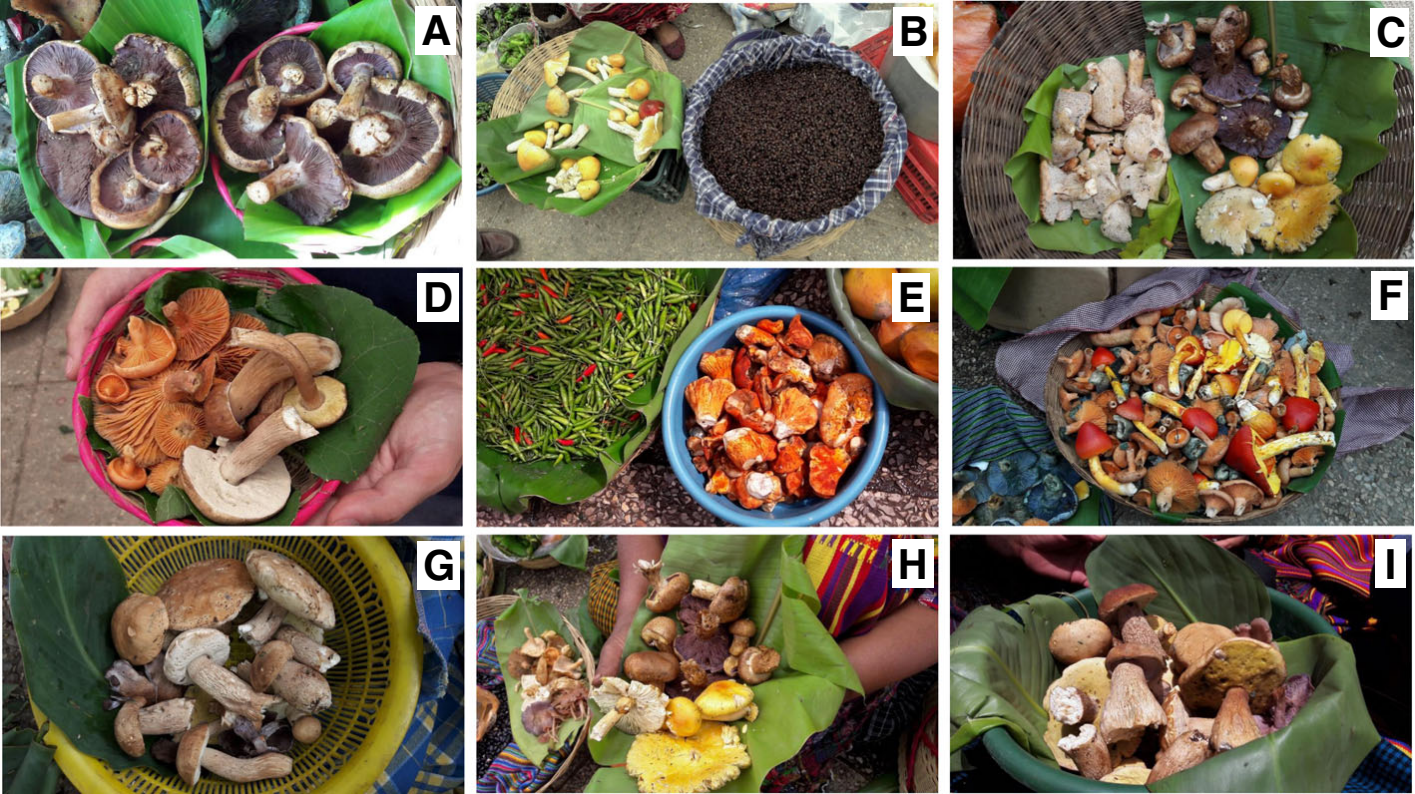
Some of the most common species of edible mushrooms that were found in the San Juan Sacatepéquez market during the study period are shown. **a** *Cortinarius* sp., *Jolom utiw* (coyote head, coyote). **b** *zompopos de Mayo* (ants of genus *Atta*) offered side-by-side with *Amanita basii* (and one *A*. *jacksonii*). **c** *Hydnum repandum*, *Cortinarius* sp., and *A*. *basii*. **d** *Lactarius deliciosus s*.*l*. and *Boletus* aff. *variipes*. **e** *Hypomyces lactifluorum* with *Russula delica*, and green local hot-peppers. **f** A basket with fresh fruit bodies of *A*. *jacksonii* (*Hongo de San Pedro*), *Lactarius deliciosus s*.*l*., and *L*. *indigo* (*Xara azul*). **g** *Boletus* aff. *variipes* and *Cortinarius* sp. **h** Local woman selling *A*. *basii*, *Cortinarius* sp., *L*. *deliciosus s*.*l*., *Lepista* aff. *sordida*, *Laccaria* aff. *laccata*, *H*. *repandum*. **i** Basket of *pancitas*, *Boletus* spp. (note the strong reticulated stipe in the large young fruit bodies) and *Lepista*

Taxonomic identification of the mushrooms sold in the San Juan Sacatepéquez market permitted to ascertain that over 40 species and varieties of edible mushrooms were commercialized during the study period. Several edible species are undescribed and are currently under taxonomic study. As discussed below, these included species that were not previously reported for Guatemala, and new reports of edible fungi for the country, like *Cortinarius* aff. *violaceus*,^1^ *Gastropila* aff. *fumosa*, *Leccinum rugosiceps*, and *Tylopilus* aff. *badiceps*. It was also possible to determine that species complexes exist for *Amanita caesarea* (including fruit bodies with yellow or orange cap and with white or yellowish gills), *Boletus variipes* (different types of reticulum of the stem and structure of the cuticle of the cap), and *Cantharellus cibarius* (variations in the coloring of the cap and structure of the hymenium). Work is undergoing to solve these complexes with the use of molecular tools. Four more *Cortinarius* species were found in the market that could not be identified to the species level because of the complexity of the genus and because they were mostly young specimens. These were clearly differentiated by the color of the gills (beige, lilac, and deep-purple), as well as by the reddish-brown or yellow pileus. Table 2 presents a list of the species observed and acquired in the study period, with 37 species and varieties recorded in 2012, 39 species and varieties recorded in 2015, 33 species in 2016 and 21 in 2017. Some of the most common species of edible mushrooms that were found in the market during the study period are shown in Fig. 2.

**Table 2:** List of the mushroom species observed and acquired in the 4 years of the study at the San Juan Sacatepéquez market

Species	2012	2015	2016	2017	Voucher #°
*Amanita basii* Guzmán & Ram.-Guill.	x	x	x	x	MICG-5017
*Amanita caesarea* (Scop.) Pers.	x	x	x	x	MICG-5016
*Amanita jacksonii* Pomerl.	x	x	x	x	MICG-4892
*Amanita* aff. *jacksonii* Pomerl.	x	x	x	x	MICG-4935
*Boletus* aff. *atkinsonii* Peck	x	x	x	x	MICG-3887
*Boletus* aff. *luteoloincrustatus* R. Flores & Simonini	x	x	x		MICG-4841
*Boletus* aff. *variipes* Peck*	x	x	x	x	MICG-4916
*Butyriboletus* sp. nov.*	x		x		MICG-3892
*Cantharellus cibarius* Fr.	x	x	x	x	MICG-5590
*Cantharellus* aff. *cibarius* Fr.	x	x			MICG-5013
*Cantharellus lateritius* (Berk.) Singer	x	x	x	x	MICG-5166
*Cantharellus* aff. *lateritius* (Berk.) Singer		x	x		MICG-5168
*Cantharellus* sp.		x			MICG-5221
*Cortinarius* aff. *violaceus* (L.) Gray*	x	x	x		MICG-4901
*Cortinarius* sp. 1*	x	x	x	x	MICG-4837
*Cortinarius* sp. 2*	x	x	x		MICG-4905
*Cortinarius* sp. 3*	x	x	x		MICG-6249
*Cortinarius* sp. 4*		x			MICG-6250
*Gastropila* aff. *fumosa* (Zeller) P. Ponce de León*		x			MICG-4805
*Helvella crispa* (Scop.) Fr.	x	x		x	MICG-2782
*Hydnum repandum* L.	x	x	x	x	MICG-6225
*Hydnum repandum* var. *album* (Quél.) Rea	x	x	x		MICG-5167
*Hydnum umbilicatum* Peck	x	x	x		MICG-5178
*Hydnum* aff. *umbilicatum* Peck	x	x			MICG-5179
*Hygrophorus russula* (Schaeff. ex Fr.) Kauffman		x	x		MICG-6233
*Hygrophorus sordidus* Peck		x		x	MICG-6234
*Hypomyces lactifluorum* (Schwein.) Tul. & C. Tul.	x	x	x	x	MICG-4824
*Laccaria amethystina* Cooke		x			MICG-5196
*Laccaria* aff. *laccata* (Scop.) Cooke	x		x	x	MICG-5180
*Lactarius deliciosus s*.*l*. (L.) Gray	x	x	x	x	MICG-4900
*Lactarius indigo* (Schwein.) Fr.	x	x	x	x	MICG-4844
*Lactarius* aff. *subpurpureus* Peck*		x	x	x	MICG-4899
*Leccinum* aff. *rugosiceps* (Peck) Singer	x				MICG-5162
*Lepista nuda* (Bull.) Coke	x	x	x	x	MICG-4700
*Lepista* aff. *sordida* (Schumach.) Singer	x	x	x	x	MICG-5186
*Ramaria araiospora* Marr & D.E. Stuntz	x		x		MICG-4911
*Ramaria* aff. *botrytis* (Pers.) Bourdot	x		x		MICG-3619
*Ramaria* aff. *flava* (Schaeff.) Quél.	x				MICG-5195
*Russula delica* Fr.	x	x			MICG-4701
*Russula* aff. *olivacea* (Schaeff.) Fr.	x	x	x	x	MICG-4904
*Russula* aff. *pulchra* Burl.	x	x			MICG-3902
*Russula virescens* (Schaeff.) Fr.	x	x	x	x	MICG-4910
*Sarcodon* aff. *squamosus* (Schaeff.) Quél.	x	x	x		MICG-5190
*Suillus* aff. *salmonicolor* (Frost) Halling*	x	x	x		MICG-4890
*Tricholoma* aff. *flavovirens*			x		MICG-5192
*Tylopilus* aff. *badiceps* (Peck) A.H. Sm. & Thiers*	x	x			MICG-4906

°A representative voucher kept at MICG (Micoteca Rubén Mayorga Peralta, Universidad de San Carlos de Guatemala) is reported

*First record as edible species for Guatemala

The number of edible mushroom species for sale in San Juan Sacatepéquez found during this study is significantly elevated, if compared with that recorded in markets by previous works conducted in Guatemala [26–30]. Surveys carried out in the departments of Chimaltenango (Tecpán, San Juan Comalapa, San Martín Jilotepeque, and Chimaltenango) and Totonicapán recorded 31 and 22 species, respectively [24, 31, 32]. When the entire country is considered, recent reviews estimate that at least 100 species are consumed traditionally in Guatemala and sold in local markets or along roadsides, especially in the highlands [15, 24, 33]. The large number of species recorded during the present investigation may be due to several factors, such as a greater number of visits to the market, better study design, and closer collaboration with local entities that facilitated contact with vendors and collectors, increase in the consumption of edible fungi and of their sale in the local market and in the country. The results obtained show that it is feasible to find a greater number of species if it is possible to carry out a greater number of samplings, even in periods of the year that are not particularly ideal for mushroom growth, such as May or November. A quick analysis of the diversity found in 2012 and 2015 shows that the month with the greatest variety of mushrooms on sale is June (data not shown). However, there have been other months with significant sale and diversity of fungi due to climatic alterations and effects of natural phenomena, such as the tropical depression 2-E in May 2012, the tropical storm Ernesto in August and the increase in rainfall in November 2015, which allowed the highest sale of *anacate* (*C*. *cibarius* and *C*. *lateritius*) in a season that is not usually ideal for mushrooms. Overall, *Amanita*, *Boletus*, *Cantharellus*, *Cortinarius*, *Hydnum*, *Lactarius*, *Ramaria*, and *Russula* are the genera that are commercialized most frequently to date, confirming previous observations, particularly those of Sommerkamp [26]. Out of 46 species recorded on sale, some 24 (52%) were also collected in the field (see below).

**Fig. 3:**
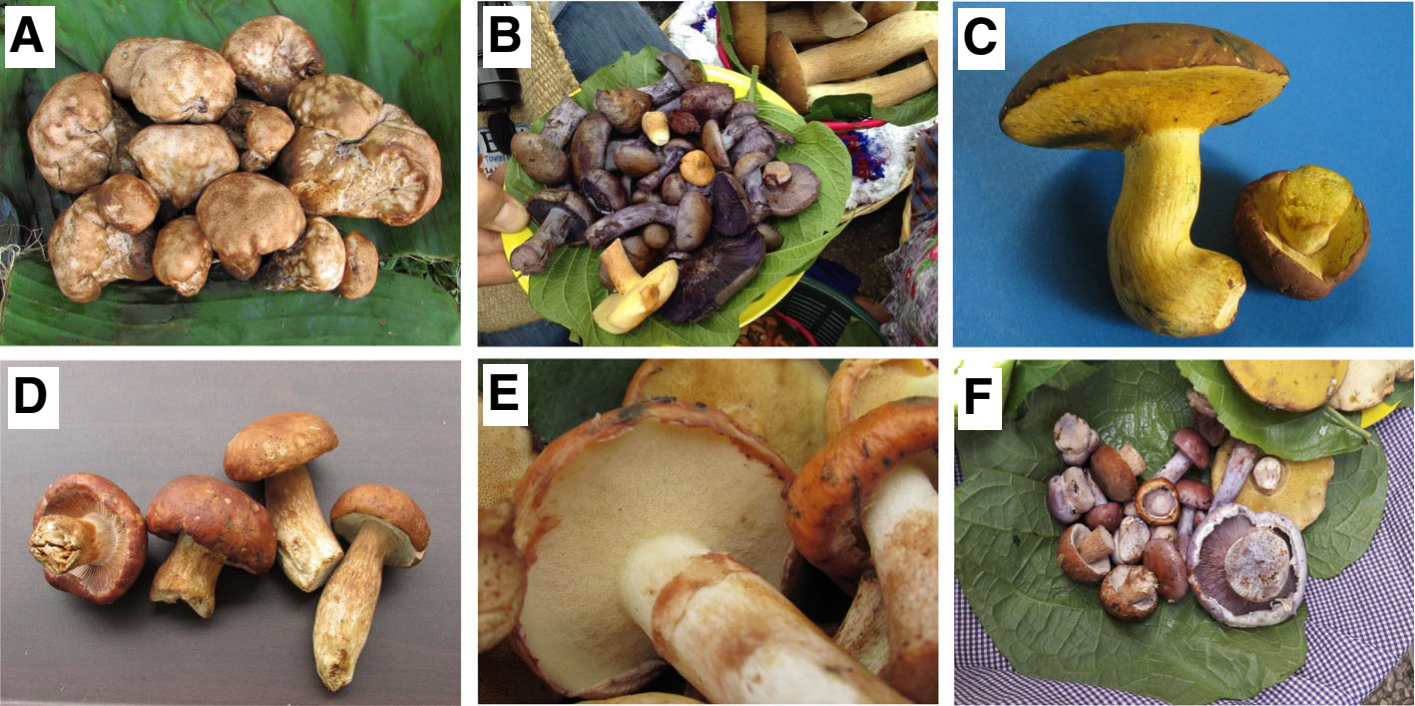
Some of the records of edible species new for Guatemala found during this study. **a** *Gastropila* aff. *fumosa*. **b** *Cortinarius* aff. *violaceus*. **c** *Butyriboletus* sp. nov. **d** Young fruit bodies of *Cortinarius* sp. and *Tylopilus* aff. *badiceps* sold at the market. **e** *Suillus* aff. *salmonicolor*. **f** *Cortinarius* spp. and *Boletus* aff. *variipes*

Several new records of edible species for Guatemala were found during this study, for example, *Gatropila* aff. *fumosa* (Fig. 3). Although years ago we learned about the consumption of a pinkish-yellowish species of *Gastropila* or *Calvatia* by some people from Pachalum, Quiché, which they call *pumpush* (unpublished data), this is the first time that the sale for consumption of a member of this genus is documented in Guatemala. The edibility of *G*. *fumosa* is unknown according to Desjardin et al. [34]. However, in Chiapas, Mexico, *Calvatia cyathiformis* (Bosc) Morgan and *Calvatia gigantea* (Batsch) Lloyd are considered edible and have medicinal uses when they are young and “when they are white” [35]. Kuo [21] mentions *C*. *gigantea* as edible in North America. Our findings confirm that among the Kaqchikel unusual mushroom species are sometimes consumed. Some years back, Morales and colleagues reported about the ascomycete *Daldinia fissa* Lloyd, sold as an edible fungus in Tecpán, Chimaltenango, where it is known in the local Kaqchikel dialect as *tzan tz*’*i* which means “dog nose,” alluding to the form of ascostroma [36].

*Tylopilus* aff. *badiceps* is an interesting finding in the market because *Tylopilus* is a genus whose species are generally not edible due to their bitter taste (Fig. 3). The largest number of *Tylopilus* species is found in North America [20]. Our species was found in the month of June 2012 and 2015 and has a great resemblance to young specimens of *Boletus edulis* Bull. and *B*. *variipes* but with redder tones, whitish hymenium and fine white reticulum on a beige background. It can be identified in the field by making cuts of the fruiting bodies and observing beige tubules with a slight grayish hue and a slightly bitter taste that disappears soon, so it can easily be confused with *Boletus* specimens. The microscopic analysis of the cuticle of the pileus confirmed a structure of the genus *Tylopilus*. The macro and microscopic analysis of the local specimens coincided enough to identify it with *T*. *badiceps* although the stipe is much clearer than reported by Bessette et al. [20] and it could be another species, which will be clarified by means of molecular tools. *Butyriboletus* sp. nov., with a bright yellow hymenium when mature, was found in 2012 in a basket of several fungi so it could be confused with other *pancitas* (*Boletus* sensu lato). This yellowish species, which produces specimens with caps of more than 10 cm in diameter (Fig. 3), was also located in an oak stand and was the first record of the genus for the country. In 2018 the same species was found in the southeastern area of Guatemala also in association with local oaks. *Leccinum rugosiceps* is a species similar to *Boletus variipes*, especially in young stages, and was found in a small basket with several other boletales. The vendor was consulted and said that it was edible and that it was called *pancita* like the rest. This species is considered edible by Kuo [21] and is relatively frequent in oak forests but had never been found on sale.

*Cortinarius* aff. *violaceus*, a new species, is another novel report of edible fungi for Guatemala. The majority of specimens were immature with blueish-violet gills but dark purple-violet to purple-brown gills when mature (Fig. 3). In 2015, the first sale of this species was identified in a small basket, besides other baskets of *Cortinarius* and other mushrooms species. This fungus can be confused at first glance with fresh and thick specimens of *Lepista sordida*, an edible species. Kuo [21] notes that the consumption of all species of *Cortinarius* should be avoided, and Desjardin et al. [34] pointed out that many species in this large genus are considered toxic and very few are edible and consumed in Europe and North America. Currently, there are no studies on the chemical composition of the 'edible' *Cortinarius* in Guatemala and their effect on human health is unknown, so it becomes a subject of biochemical research and public health interest.

We also strived to identify other edible mushroom species that are collected and consumed in San Juan Sacatepéquez and that are not sold in the municipal market. It was found that unlike other places in Guatemala, collectors only consume the species they sell in the market. However, two vendors and three collectors mentioned *Pseudofistulina radicata*, known as *azadón* (hoe) or *hongo de guachipilín*, for their own consumption. Another collector referred to *Sebacina sparassoidea* (Lloyd) P. Roberts,  known as *kenk*’*x* or *moco* or *baba* (“mucus,” “saliva”), and two collectors mentioned that there are people who collect red and pink specimens of *Russula* spp., which they call *Curé*, in reference to a reddish local bird. Apart from the local wild mushrooms, it is now possible to encounter occasional sales of imported mushrooms, a product that is gaining ground nationally. Although *Pleurotus*, the oyster mushroom, is a fungus that is grown in rural areas and sold in some municipal markets in Guatemala, only one local producer was found in this area who grows it for its own family consumption.

**Fig. 4:**
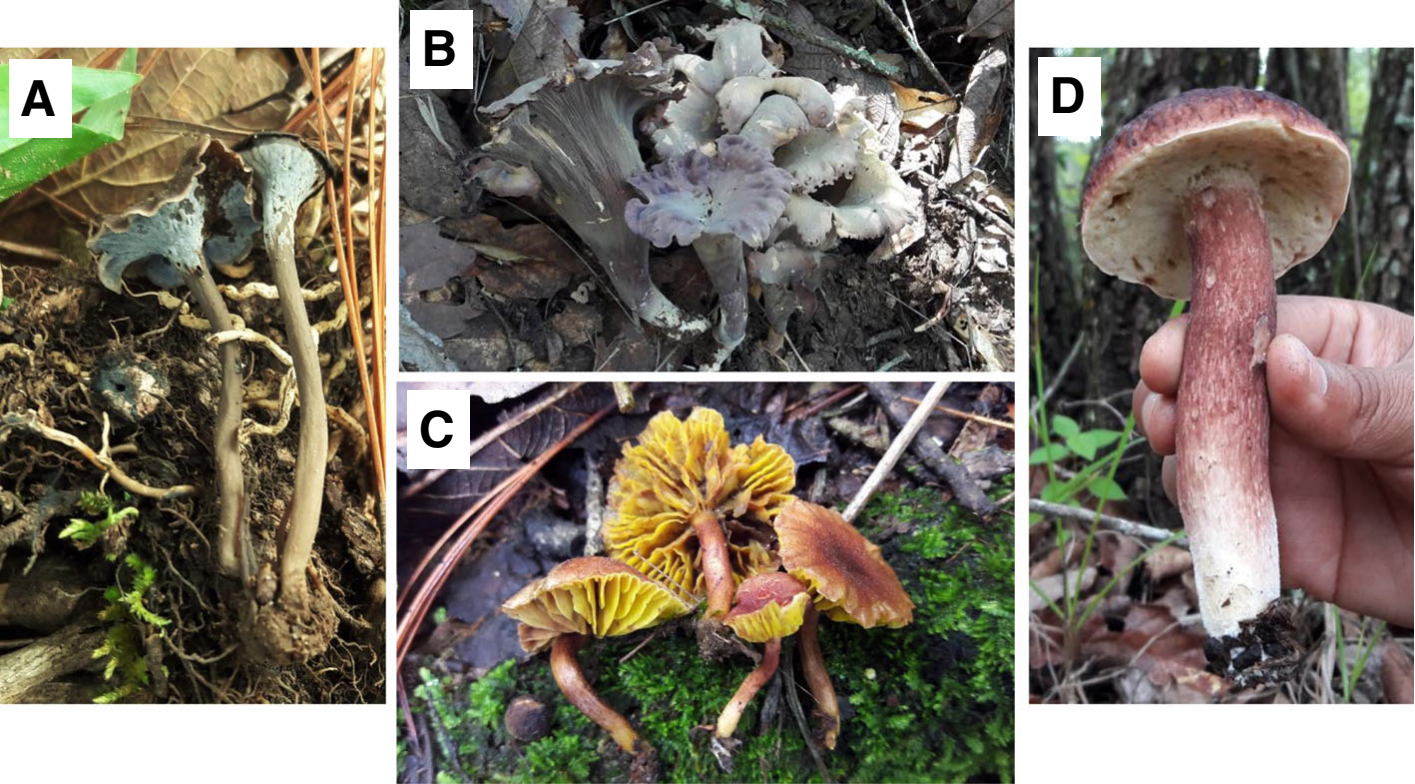
Some interesting, new species records for Guatemala are shown. **a** *Pseudocraterellus calyculus*. **b** *Gomphus* aff. *clavatus*. **c** *Phylloporus* sp. **d** *Xanthoconium* aff. *separans*. All depicted species are from field samplings (see Table 3)

Since the variety of edible mushrooms sold at the market depends strictly on the diversity of the macrofungal contingent in the area surrounding San Juan Sacatepéquez, where edible mushrooms are searched for and collected, we conducted a preliminary survey of the macrofungal species present in pine-oak forests of the municipality (see Additional file 1: Figure S1 for a map of the forested areas of the Municipality of San Juan Sacatepéquez). The field trips were interesting also from a strictly ethnomycological point of view: under these circumstances, we have witnessed fundamental aspects of the transmission of mycological knowledge, such as the way of selecting specimens is taught and specific places where mushrooms appear every year are indicated, a knowledge that has passed from generation to generation. Some male collectors said that many collectors do not share their knowledge with people outside their family because they think that if more people would know that information, their mushrooms collection and/or income could be affected. Table 3 lists the 100 species of wild mushrooms that were found, including edible and inedible, mycorrhizal and saprophytic, in field samplings carried out in the company of some collectors and Cementos Progreso personnel, from 2012 to 2017. During these forays, species new for the country were recorded, and some likely undescribed species found. Some interesting, new species records for Guatemala are shown in Fig. 4.

**Table 3:** Wild mushrooms collected in pine-oak forests in the Municipality of San Juan Sacatepéquez from 2012 to 2017

Species	2012	2015	2016	2017	Voucher #°
*Agaricus* sp.			x	x	MICG-6487
*Amanita* aff. *atkinsoniana* Coker				x	MICG-6379
*Amanita basii* Guzmán & Ram.-Guill.	x	x	x	x	MICG-5625
*Amanita* aff. *ceciliae* (Berk. & Broome) Bas			x	x	MICG-6488
*Amanita* aff. *citrina* Pers			x	x	MICG-6498
*Amanita jacksonii* Pomerl.	x	x	x	x	MICG-2837
*Amanita* aff. *apntherina* (DC.) Krombh.	x				MICG-2426
*Amanita* aff. *rubescens* Pers.			x	x	MICG-6490
*Amanita* aff. *vaginata* (Bull.) Lam.	x				MICG-1802
*Amanita* aff. *verna* (Bull.) Lam.	x				MICG-2491
*Aureoboletus russellii* (Frost) G. Wu & Zhu L. Yang				x	MICG-6703
*Auricularia auricula-judae* (Bull.) Quél.	x				MICG-2492
*Boletus luteoloincrustatus* R. Flores & Simonini			x		MICG-5485
*Boletus* sp. nov. 1*			x	x	MICG-5148
*Boletus* sp. nov. 2*			x	x	MICG-5557
*Butyriloboletus* sp. nov.*				x	MICG-5901
*Calvatia* sp.			x		MICG-5240
*Camarophyllus* sp.	x				MICG-2493
*Cantharellus cibarius* Fr.		x	x	x	MICG-5285
*Cantharellus* aff. *confluens* (Berk. & M.A. Curtis) R.H. Petersen			x		MICG-5286
*Cantharellus lateritius* (Berk.) Singer	x	x	x	x	MICG-5266
*Chroogomphus* aff. *rutilus* (Schaeff.) O.K. Mill.			x		MICG-6006
*Clavaria argillacea* Pers.	x				MICG-2794
*Clavulina cinerea* (Bull.) J. Schröt			x	x	MICG-5177
*Cordyceps militaris* (L.) Fr.	x				MICG-2462
*Coriolus* sp.	x				MICG-2096
*Cortinarius sanguineus* (Wulfen) Gray	x	x			MICG-2500
*Cortinarius violaceus* (L.) Gray				x	MICG-6705
*Cortinarius* sp. 1*				x	MICG-6243
*Cortinarius* sp. 2*				x	MICG-6244
*Cortinarius* sp. 3*				x	MICG-6246
*Cortinarius* sp. 4*				x	MICG-6247
*Cotylidia* sp.	x				MICG-2088
*Craterellus fallax* A.H. Sm.			x	x	MICG-6666
*Craterellus* aff. *lutescens* (Fr.) Fr.			x		MICG-6672
*Craterellus tubaeformis* (Fr.) Quél.	x		x	x	MICG-4525
*Dermocybe* sp.			x	x	MICG-5500
*Entoloma murrayi* (Berk. & M.A. Curtis) Sacc.		x	x		MICG-5187
*Entoloma* sp.				x	MICG-6496
*Favolus tenuiculus* P. Beauv.		x	x		MICG-5510
*Geastrum* sp.			x	x	MICG-6499
*Gliophorus psittacinus* (Schaeff.) Herink*		x			MICG-3173
*Gomphus* aff. *clavatus* (Pers.) Gray*			x	x	MICG-5461
*Grifola frondosa* (Dicks.) Gray			x		MICG-5235
*Gymnopus dryophilus* (Bull.) Murrill			x	x	MICG-6495
*Helvella crispa* (Scop.) Fr.			x		MICG-5515
*Helvella* aff. *lacunosa* Afzel.		x	x	x	MICG-5194
*Helvella macropus* (Pers.) P. Karst.	x				MICG-2802
*Helvella* sp. nov.		x		x	MICG-3174
*Hydnum repandum* L.	x	x	x	x	MICG-6227
*Hortiboletus* sp. nov. 1*			x		MICG-5305
*Hortiboletus* sp. nov. 2*			x		MICG-5703
*Hygrocybe* aff. *coccineus* (Schaeff.) P. Kumm.		x			MICG-2885
*Hygrocybe* aff. *flavescens* (Kauffman) Singer	x				MICG-1905
*Hygrophorus* aff. *sordidus* Peck			x		MICG-5234
*Hygrophorus* sp.	x				MICG-2234
*Hypholoma* sp.			x		MICG-4326
*Hypomyces* aff. *luteovirens* (Fr.) Tul. & C. Tul.			x		MICG-5175
*Inonotus* sp.	x				MICG-2356
*Laccaria laccata* (Scop.) Cooke	x	x			MICG-3180
*Lactarius* aff. *areolatus* Hesler & A.M. Sm.*		x			MICG-3912
*Lactarius chrysorrheus* Fr.	x	x	x	x	MICG-1961
*Lactarius deliciosus s*.*l*. (L.) Gray	x		x	x	MICG-6251
*Lactarius indigo* (Schwein.) Fr.	x	x	x	x	MICG-6252
*Lactarius psammicola* A.H. Sm.		x	x	x	MICG-5224
*Lactarius rimosellus* Peck	x	x			MICG-2884
*Lactarius* aff. *subplintogalus* Coker	x				MICG-2442
*Lactarius* aff. *subpurpureus* Peck		x	x		MICG-4835
*Lactarius* aff. *yazooensis* Hesler & A.H. Sm.				x	MICG-6268
*Leccinum* aff. *rugosiceps* (Peck) Singer		x			MICG-3424
*Lentinula* aff. *boryana* (Berk.) & Mont.) Pegler*		x			MICG-2999
*Lentinus* sp.		x			MICG-3185
*Lepista* aff. *sordida* (Schumach.) Singer		x	x	x	MICG-5275
*Lycoperdon perlatum* Pers.		x	x	x	MICG-6508
*Marasmius* sp.		x	x		MICG-5609
*Ophiocordyceps melolonthae* (Tul. & C.Tul) G.H. Sung, J.M. Sung, Hywel-Jones & Spatafora			x		MICG-5231
*Peziza* aff. *phyllogena* Cooke*		x			MICG-3189
*Peziza* sp.			x		MICG-5189
*Phlebopus* sp.	x				MICG-2153
*Phylloporus* sp.*	x	x			MICG-3450
*Phyllotopsis nidulans* (Pers.) Singer		x			MICG-3164
*Pisolithus arhizius* (Scop.) Rauschert		x	x	x	MICG-6271
*Pseudocraterellus calyculus* (Berk. & M.A. Curtis) D.A. Reid*			x	x	MICG-5226
*Pseudofistulina radicata* (Schwein.) Burds.	x		x		MICG-5511
*Pulveroboletus ravenelii* (Berk. & M.A. Curtis) Murrill	x		x		MICG-5551
*Ramaria botrytis* (Pers.) Bourdot	x				MICG-2908
*Russula* aff. *cyanoxantha* (Schaeff.) Fr.	x	x			MICG-2512
*Russula delica* Fr.	x				MICG-2713
*Russula* aff. *foetens* Pers.	x		x	x	MICG-6245
*Russula grata* Britzelm.	x		x		MICG-4902
*Scleroderma polyrhizum* (J.F. Gmel.) Pers.	x	x	x	x	MICG-6514
*Sebacina* aff. *schweinitzii* (Peck) Oberw.	x	x			MICG-3566
*Sparassis* aff. *spathulata* (Schwein.) Fr.		x	x		MICG-4591
*Suillus* aff. *punctipes* (Peck) Singer	x				MICG-2561
*Suillus* aff. *salmonicolor* (Frost) Halling		x	x		MICG-3516
*Trametes* aff. *versicolor* (L.) Lloyd	x				MICG-2991
*Tremella* aff. *mesenterica* Retz.		x			MICG-3229
*Trichoglossum hirsutum* (Pers.) Boud.	x				MICG-2230
*Tylopilus* aff. *badiceps* (Peck) A.H. Sm. & Thiers*	x				MICG-2449
*Xanthoconium* aff. *separans* (Peck) Halling & Both*	x				MICG-2892

*First record for Guatemala

°A representative voucher kept at MICG (Micoteca Rubén Mayorga Peralta, Universidad de San Carlos de Guatemala) is reported

The first reports from field samplings include *Cantharellus* aff. *confluens* and other species close to *C*. *cibarius*, several boletales such as *Xanthoconium* aff. *separans*, a reddish-purple fungus, very scarce and edible, reported only in the eastern zone of North America and Mexico [21]. *Butyriboletus* sp. nov., with a brown cap and bright yellow hymenium in mature specimens, a genus never reported from Central America before. *Phlebopus* sp., possibly a new member of an interesting genus which counts several species in the Neotropics [37]; although it is usually believed to be a saprobic fungus, our specimen was found in a mixed pine-oak forest. *Pulveroboletus ravenelii* is a unique yellow-colored fungus that has been found in pine and pine-oak woods in warm areas and at lower altitudes in Guatemala, but which also exists relictually and disjointly in other countries of the Americas, and even in Australia [38]. *Phylloporus* also has at least one local small species that could not be identified to species level; it was distinct from those reported in South America, where the genus seems to be more abundant [39]. *Gomphus* aff. *clavatus* is another new record for Guatemala; we found fruit bodies in 2016 in a mixed forest close to Cruz Blanca, where the basidioma were observed for more than a month, developing large ‘trumpets’. Other frequent fungi were *Scleroderma* and *Pisolithus* that could be used as a good source of mycorrhizal inoculum for pine and oak plants; local collectors do not give them any use even though in other regions of the country—as the close San Martín Jilotepeque—these mushrooms are used as healing agents [32]. *Pisolithus* is called *frog’s mushroom* in a depictive manner. Other mycorrhizal fungi of great ecological value encountered in field samplings were *Laccaria laccata*, *Suillus* spp., and *Peziza* spp. It was interesting to find four species of *Helvella*, including a new species that is currently being described (Fig. 4). A genus that was not frequent in the forests around San Juan Sacatepéquez was *Ramaria*, a situation contrary to what is found in other pine-oak forests towards the western zone of Guatemala, even at the close Alux mountainous system, where many large species grow.

### Selling practices and preparation methods

**Fig. 5:**
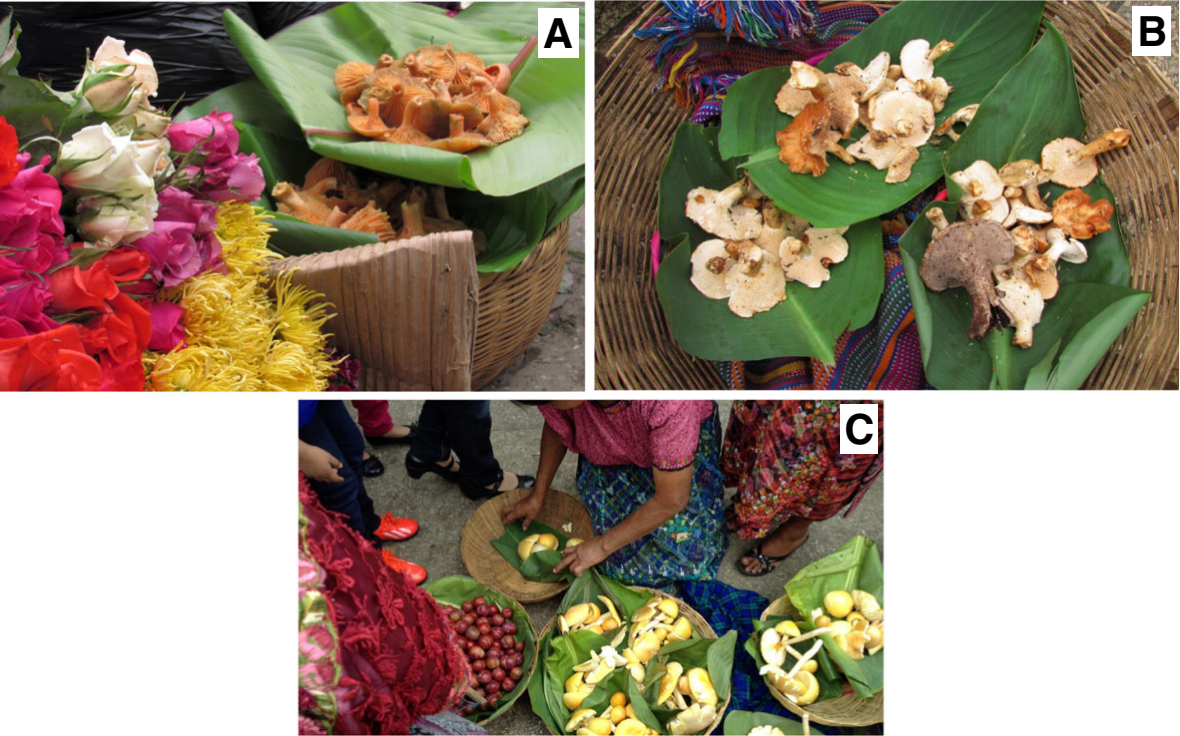
Presentation of mushrooms for selling at the San Juan Sacatepéquez market. **a** *Lactarius* sold on fresh banana leaves. San Juan Sacatepéquez market is also famous for the trade of flowers. **b** *Hydnum* and *Sarcodon* on leaves of maxán (*Calathea lutea* and *C*. *insignis*, Marantaceae). **c** Fresh *Amanita basii* offered in leaf-lined baskets

The edible mushrooms in San Juan Sacatepéquez market come, with few exceptions, from mixed forests of pine (*Pinus pseudostrobus*, *P*. *oocarpa*) and oak (*Quercus brachiystachis*, *Q*. *peduncularis*, *Q*. *tristis*) located in areas that belong to a village, hamlet, or community of the municipality (see Additional file 1: Figure S1). The locations, that were most mentioned by sellers were Cruz Blanca, El Pilar I, El Pilar II, and Comunidad de Ruiz (Fig. 1). The commercialization of the mushrooms, used only as food by the residents, is done exclusively in the municipal market, where the collectors bring the mushrooms in baskets to sell by themselves or sell the mushrooms to other vendors (retailers). No effort is made to preserve mushrooms, which are all sold/purchased fresh. In the process of supply and resale, it was observed that the buyers select their mushrooms and readjust them according to their experience. The fresh mushrooms are placed in baskets on leaves of maxán (*Calathea lutea* and *C*. *insignis*, Marantaceae) or banana leaves to keep them moist and prevent their decomposition (Fig. 5). Some vendors place mushrooms in pieces of local cloth to cover and protect them from the sun and flies; others place mushrooms in plastic or even clay containers. Occasional collectors, on the other hand, take their mushrooms (usually just a few specimens) directly to the market, either in plastic bags or in wrapped baskets and expose them for sale; these mushrooms are quickly acquired by other vendors or local consumers, generally at lower prices than those sold by experienced vendors. Some mushrooms are sold in small *medidas* (portions) of the same species or group of similar species, such as the *A*. *caesarea* complex, *Boletus variipes* group, *C*. *cibarius/C*. *lateritius*, *Lactarius deliciosus s*.*l*., *L*. *indigo*, *Russula* spp., and *Suillus* spp. Others can be found mixed, as is the case with *Helvella*, *Hydnum*, *Hygrophorus*, *Laccaria*, *Ramaria*, *Russula*, and *Sarcodon*, or the case of the boletales *Boletus*, *Butyriboletus*, *Suillus*, and *Tylopilus*. Some mushrooms are marketed by pound, half pound, and even as a single carpophore. The most significant ones, sold per pound, are the *anacates* (*C*. *cibarius* and *C*. *lateritius*) irrespective of the abundance. Table 4 lists the species that are sold more frequently in the San Juan Sacatepéquez market, the way they are marketed, and if sold alone or accompanied by other fungal species.

**Table 4:** List of mushroom species that are sold more frequently in the San Juan Sacatepéquez market, the way they are marketed, and if sold alone or accompanied by other fungal species

Species	Selling unit	Selling specificity
*Amanita caesarea* complex	Single, portion, basket	Alone/with other spp.
*Amanita jacksonii*	Portion, basket	Alone/with other spp.
*Boletus* spp. (porcini group)	Single, portion, basket	Alone/with other spp.
*Gastropila* aff. *fumosa*	Basket	Alone
*Cantharellus cibarius*	Pound, half pound	Alone or with *C*. *lateritius*
*Cantharellus lateritius*	Pound, half pound	Alone or with *C*. *cibarius*
*Cantharellus* sp.	Pound	Alone
*Cortinarius* spp.	Portion	Alone/with other spp.
*Helvella crispa*	Portion	With other spp.
*Hydnum repandum*	Portion	Alone/with other spp.
*Hydnum repandum var*. *album*	Portion	With other spp.
*Hydnum umbilicatum*	Portion	Alone/with other spp.
*Hygrophorus russula*	Portion	With other spp.
*Hygrophorus sordidus*	Portion	With other spp.
*Hypomyces lactifluorum*	Portion, basket	Alone
*Laccaria* spp.	Portion	With other spp.
*Lactarius deliciosus s*.*l*.	Portion, basket	Alone or with other *Lactarius*
*Lactarius indigo*	Portion, basket	Alone/with other spp.
*Lactarius* aff. *subpurpureus*	Portion	Alone or with other *Lactarius*
*Leccinum* aff. *rugosiceps*	Portion	With other spp.
*Lepista nuda*/*L*. aff. *sordida*	Basket	Alone/with other spp.
*Ramaria* spp.	Portion	With other spp.
*Russula delica*	Portion, basket	With other spp.
*Russula* aff. *olivacea*	Basket	Alone or with other *Russula*
*Russula* aff. *pulchra*	Portion	With other spp.
*Russula virescens*	Basket	With other spp.
*Sarcodon* aff. *squamosus*	Portion	With *Hydnum* spp.
*Suillus* aff. *salmonicolor*	Portion, basket	Alone or with other *pancitas*
*Tylopilus* aff. *badiceps*	Portion	With other spp.

The price of mushrooms in the market varies according to the season and abundance. The best-selling months are June and September, which correspond to the months of greatest precipitation in the region. Little price variation was found when comparing the 2 years, 2012 and 2015. Table 5 shows the sale prices (in the local currency, Guatemalan quetzal; 1.00 US$ = Q7.7) at which the mushrooms are sold. During the visits to the market, in the period of study, it was observed that *A*. *caesarea* complex (including *A*. *basii* and *A*. *jacksonii*) commonly known as *Hongos de San Juan*, are the mushrooms with the highest sale price due to their demand and relatively short season of growth (June and sometimes August–September). These mushrooms are offered by 'basket' (*canasto*), which can get to cost up to Q200.00 (US$ 26.00) each or by *medida* (Q30.00, US$ 4.00), where the basket can contain approximately between 5 and 6 pounds as reported by the interviewed vendors, while a portion can contain from 3 to 12 fruit bodies, depending on the size and freshness of the fungi. The reddish-orange *A*. *jacksonii* is usually marketed by portion, with a content of 4 to 5 fruit bodies and an average price of Q25.00 (US$ 3.25), a little lower than that of the yellow *A*. *basii*. Some interviewed buyers told us they avoid *A*. *jacksonii* because its color resembles that of *A*. *muscaria*, and this spurs some doubt about safety and edibility. Indeed, during this study, it was found that some people who have consumed raw *A*. *jacksonii* may feel dizzy and this of course influences their future purchases. As a result of these considerations, yellow amanitas always sell faster. It is important to point out that *A*. *muscaria* has not been found in the area but is frequent in pine forest around 2000–3000 mts in the central-western zones of Guatemala.

**Table 5:** Sale prices (in local currency, Guatemalan quetzal, 1.00 US$ = Q7.7) at which the mushrooms were sold, as recorded in 2012 and 2015

Species	2012	2015	Selling unit
*Amanita caesarea complex*	Q30.00–100.00	Q35.00–200.00	Portion, basket
*Amanita jacksonii*	Q25.00	Q30.00–35.00	Portion
*Boletus* spp. (porcini group)	Q5.00	Q20.00	Single, portion
*Cantharellus cibarius/C*. *lateritius*	Q10.00–40.00	Q20.00–45.00	Portion, pound
*Cortinarius* spp.	Q8.00	Q10.00–25.00	Portion
*Gastropila* aff. *fumosa*	–	Q10.00–20.00	Portion
*Hydnum* spp.	Q10.00	Q10.00	Portion
*Lactarius deliciosus s*.*l*.	Q20.00–25.00	Q10.00–20.00	Portion
*Lactarius indigo*	Q10.00–20.00	Q10.00–20.00	Portion
*Lactarius* aff. *subpurpureus*	Q10.00	Q10.00	Portion
*Lepista nuda*/*L*. aff. *sordida*	Q8.00–10.00	Q8.00	Portion
*Russula* spp.	Q10.00	Q10.00	Portion
*Sarcodon* aff. *squamosus*	Q10.00	Q10.00	Portion
*Suillus* spp.	Q10.00	Q10.00	Portion

*Cantharellus cibarius* and *C*. *lateritius* (*anacates*) also have high prices, and although they are usually sold per pound, they can be sold for half a pound and even by portion in the first fruiting. Prices range between Q45.00 (US$ 6.00) per pound and Q10.00 (US$ 1.3) per portion. Generally, *anacates* sold in the San Juan Sacatepéquez market are always fresh and are often sent to markets in Guatemala City. *Lactarius deliciosus s*.*l*., *L*. *indigo* and *L*. aff. *subpurpureus* are sold by portion, alone, or mixed with other fungi, at much lower prices than *Amanita* and *Cantharellus*, between Q10.00 and Q25.00 (US$ 1.3–3.25), a price that can vary according to the freshness of the mushroom, which is reflected in the buyer’s eye by the intensity of the color (orange, blue, or pink). This genus is very useful to mushroom sellers because its abundance serves to create volume in sales. Mushrooms of the *Boletus variipes* group (*B*. *variipes*, *B*. *luteoloincrustatus*, and *Boletus* spp.) can be purchased as a single carpophore or per portion, which may contain a few specimens. Their price is low and ranges from Q5.00 per fruit body to Q20.00 (US$ 2.60) per portion; in general, only young specimens are sold. A particularity noted at the market in San Juan is that bolets quickly become infested with larvae, a process that has been more evident in 2015, probably due to an increase in temperature and local humidity. It was observed that the majority of vendors cut off a good part of mushrooms’ stem, to leave the unspoilt part exposed; we also observed that some buyers demanded a price reduction while purchasing specimens with numerous holes in the *Boletus* caps. Finally, the *Cortinarius* usually fetch low prices, which range between Q8.00 and Q25.00 (US$ 1.00-3.25) per portion depending on the season, although the average is Q8.00–10.00 (US$ 1.00-1.3). This price makes the consumption of these fungi, usually offered fresh and in good conditions, more affordable than other species on sale. This genus, known as *cabeza de coyote* (coyote’s head), or simply *coyote*, from the Kaqchikel *jolom utiw*, is more expensive in the neighboring San Martin Jilotepeque, Chimaltenango, and Comalapa [32].

During the purchase of mushrooms in the market, the vendors were asked about the local preparation methods and forms of consumption of the edible fungi. We also consulted with collectors, Novella Foundation staff, women who bought mushrooms in the market, and some Cementos Progreso workers. Table 6 shows the forms of preparation reported. The recurrent recipe for most mushrooms is roasted with lime and salt, followed by frying; when roasted, mushrooms are usually consumed together with corn tortillas. This modality applies to *Amanita*, *Hydnum*, *Lactarius*, and *Ramaria*. What observed coincides with what reported for other Kaqchikel areas, such as Tecpán, Comalapa, and San Martín Jilotepeque where the majority of the surveyed population expressed a clear preference for this way of mushroom preparation [30–32]. Another frequent form of consumption of fungi is in *chirmol* (tomato and onion), where *Amanita*, *Boletus*, *Cantharellus*, *Cortinarius*, *Gastropila*, *Hypomyces*, and *Suillus* are added. The only two fungi that are used for *tamalitos* or *chuchitos* (a local small *tamal*) are *Cantharellus* (*anacate*) and *Hypomyces lactifluorum* (car horn) thanks to their fibrous-fleshy consistency. The consumption of *tamalitos* with mushrooms was also reported in Tecpán by Morales et al. [30]. Of notice, *Lepista* aff. *Sordida* is added to the preparation of bean broth, and its Spanish name (*frijolito*) refers to the blueish color of mushroom that in turn reminds of the violet color of black beans when soaked in water previous to boiling. The people surveyed explained that roasting mushrooms is the most common form of preparation for the ease of doing so both in the field and at home, since mushrooms can be placed on the *comal* (clay griddle) at the same time as preparing tortillas. All the species are still cooked following the traditional methods and many were eaten by the research group that found them highly palatable.

**Table 6:** Local preferred preparations for edible fungi in the San Juan Sacatepéquez area

Species	Preparation
*Amanita caesarea* complex	Grilled, with lime and salt, in chirmol (crushed tomato, onion, chili)
*Amanita jacksonii*	Grilled, with lime and salt, in chirmol
*Boletus* spp. (porcini group)	Grilled, fried with tomato and onion
*Gastropila* aff. *fumosa*	Tomato and onion
*Cantharellus cibarius/C*. *lateritius*	With rice, small tamales with chipilín (*Crotalaria longirostrata*), pulique (a thick meat and vegetable stew), tomato and onion
*Cortinarius* spp.	Grilled, with chirmol
*Hydnum* spp.	Grilled, with lime and salt
*Hypomyces lactifluorum*	Fried with tomato and onion, usually served in small tamales
*Lactarius deliciosus s*.*l*.	Grilled, with lime and salt
*Lactarius indigo*	Grilled, with lime and salt
*Lactarius* aff. *subpurpureus*	Grilled, with lime and salt
*Lepista nuda*/*L*. aff. *sordida*	Grilled, with lime and salt, added to black beans
*Ramaria* spp.	Grilled, with lime and salt
*Russula* spp.	Grilled
*Suillus* spp.	Fried, with tomato and onion

### Mushrooms in the Kaqchikel culture: vernacular names, traditional mycological knowledge, and associated beliefs

In the municipality of San Juan Sacatepéquez, several species of edible and non-edible mushrooms are identified with names in Kaqchikel, Spanish, or both. Table 7 reports these local names and compares these with those reported in other municipalities of the Kaqchikel area. Overall, it can be observed that there is a fair similarity in the names of the mushrooms in San Juan Sacatepéquez when compared with neighboring Kaqchikel populations. Exceptions do exist, though, like local names for *Hypomyces*, which is the most variably called mushroom in the Kaqchikel area recorded so far. In San Juan Sacatepéquez, different species of mushrooms are sold under the same common name in Spanish, a situation that changes a little when they are nominated in Kaqchikel. Both Kaqchikel and Spanish names generally refer to their color, shape (and resemblance to some animal parts), place where they grow, etc. Spanish common names include: *Hongos de San Juan*, *xaras* o *sharas*, *anacates*, *pancitas*, *cabezas de coyote* (coyote heads), *lengua de gato* (cat tongue), *lengua de venado* (deer tongue), *trompas de coche* (pig mouth), *muñequitos* (small dolls), and *cachos de venado* (deer horns). These are collective names, indicating more than one species, but generally these belong to the same scientific genus or to a closely related one, as explained below.

**Table 7:** Local names of mushrooms in San Juan Sacatepéquez and comparison with other municipalities of the Kaqchikel area

	Department							
	Guatemala		Chimaltenango				Sacatepéquez	
	Municipality							
	San Juan Sacatepéquez		San Juan Comalapa	San Martín Jilotepeque	Patzún	Tecpán	Sumpango	Santo Domingo Xenacoj
Species	Spanish	Kaqchikel						
*Amanita caesarea* complex	Tecomate amarillo/Hongo de San Juan	Q’atzuy	Q’atzuy	Q’atzuy	Q’atzuy	Q’atzuy	Q’atzuy	Q’atzuy
*Amanita jacksonii*	Hongo de San Pedro	Ruq’u San Pedro						
*Boletus edulis* group	Pancita/Palo ladino	Lix/Patün lix					Rusemit kuk	
*Cantharellus cibarius*/*C*. *lateritius*	Anacate	Q’axul/ K’axul’	Q’axul		Q’axul	Q’axul	Q’anxul	Q’axul
*Cortinarius* spp.	Cabeza de coyote	Jolom utiw	Ruwi’ utiw	Jolo utiw		Jolom utiw		Jolon utiw
*Helvella crispa*	Muñequitos		Mo’s			Numq’eq	Xikin b’ur	
*Hydnum repandum*/*H*. *r*. *var*. *album*/*H*. *umbilicatum*	Lengua de venado/lengua de gato	Raq’ mazat/raq’ mes	Raq’ mazat		Raq’ mazat	Raq’ mazat	Raq wakax	Raq’mazat
*Hypomyces lactifluorum*	Trompa de coche	Rutza’n aq	Kaqaxtën		Tzan Aq	Kaqaxän	Uparatza’n aq	
*Laccaria amethystina*/*L*. aff. *laccata*	Canilla de pajarito/cabeza de pajarito	Raqun chip/rujolom tonch’ich’	Xcampraña			Tutza’naq		
*Lactarius deliciosus s*.*l*.	Shara amarilla	Xar, Tzum cabro	Tolor	Qän Xar	Kaqix	Kaqix	Amacaria	Kaqix
*Lactarius indigo*	Xara azul	Rujolon xar	Ruwi’ Xar	Xar	Xar	Raxwach kaqix Räx kaqix Räx okox	Upawi’ xar/Xar	Raxwach kaqix
*Lactarius* aff. *subpurpureus*	Xarita anaranjada	Xaritas						
*Lepista nuda*/*L*. aff. *sordida*	Frijolito	Li’x	Panq’oq			Panq’oq		
*Ramaria araiospora/R*. spp.	Cabeza de gallo	Rujolon äk	Tzikej			Tzikej Rixk’eq Chikop		
*Suillus* aff. *salmonicolor*	Pancita	Lix	Punpun			Punpu’x		
*Tricholoma flavovirens*	Pancita esponjita	Lix	Tonch’ich’			Jolon Toch’ich’		
*Tylopilus* aff. *badiceps*	Pancita							

Source: data obtained through interviews and contacts with vendors and collectors in the frame of this study in 2012 and 2015, and compared with published information [24, 31, 32, 40]

The *Hongos the San Juan *(*A*. *caesarea* complex) include light yellow, orange-yellow, and red-capped mushrooms. Guzmán and Ramírez-Guillen [41] have published a thorough study about the diversity of this complex in Mexico and Guatemala, describing several very similar species based on microscopy analysis. According to these authors, the real *A*. *caesarea* is rare in Mexico, and probably also in Guatemala. The members of this group growing in the Guatemala highlands are currently under study through a mix of morphological and molecular tools. As for *A*. *jacksonii*, characterized by a brightly red pileus, it was found that some vendors call it *Hongo de San Pedro*, since they say it grows more towards the neighboring San Pedro Sacatepéquez. Although it is a locally frequent species, a clear preference for '*A*. *caesarea'* was found in sale and consumption. As for the names in the Kaqchikel language, '*A*. *caesarea*' is known as *Q*’*atzuy* (yellow tecomate), a name that coincides with that reported from other areas of Kaqchikel culture like Tecpán [30], Comalapa [31], San Martín Jilotepeque [32], Patzún [32], and Sumpango [40] (department of Sacatepéquez) (Table 7). *A*. *jacksonii* is known as *Ruq*’*u San Pedro*, which means mushroom of San Pedro. *A*. *caesarea* complex is the emblematic mushroom group of San Juan Sacatepéquez, to the extent that market vendors associate the term “mushroom” with these basidioma, and on the day of the patronal feast (June 24) many people come early to buy these mushrooms for consumption and for local resale, or for resale in Guatemala City and other places, as could be verified, with prices of up to Q300.00 (US$ 39.00) for a basket with more than 10 large fruiting bodies.

The *pancitas* are mainly the bolets in the genus *Boletus*, formerly *edulis* group, characterized by a hymenium with initially white and then yellow to olive-green pores, without change of color to the cut, reticulated stipe, white context, and pleasant flavor. Locally the specimens are sold in small baskets, usually one or two sister species, sometimes mixed with other fungi. Almost always young specimens are found, with the hymenium still white and immature and with the base of the stipe cut or slightly peeled with a knife, to remove the soil. Others are cut to the middle of the stem if there are spots or deterioration. The specimens observed in the market were not *B*. *edulis* but rather other species, probably two or three, close to *B*. *luteoloincrustatus*, a species described for Guatemala [42], to *B*. *atkinsonii*, *B*. *variipes*, and *B*. *nobilissimus* Both & R. Riedel from North America [20, 43], and also *B*. *quercophilus* Halling & G.M. Muell. from Costa Rica [44]. For the complete determination of local species, a deep taxonomic and phylogenetic analysis is under way in collaboration with Italian and Chinese researchers. As for the names in Kaqchikel, the *pancitas* are known by three names: *lix* (fungus), *patún* or *lix patún* (oak mushroom with dark cap), and *patún ladino* (oak mushroom with pallid cap). In interviews with Cementos Progreso staff and some vendors, the name of *patún* or *lix patún* was confirmed for those specimens associated with oak, particularly the mushrooms with the darkest pileus and stipe. The generic name of *lix* is also applied to specimens of *Suillus*, while in Comalapa and Tecpán these mushrooms are called *punpu*’*x*, which means spongy [30, 31], and *tolero* in Jilotepeque [32]. Mushrooms of the genus *Suillus* are rarely sold in Guatemalan markets. It was also found that some vendors apply the name *pancita* to a very different agaricoid basidiomycete, *Tricholoma* aff. *flavovirens*, because of the yellow color of gills.

The *sharas* or *xaras* refer to the edible mushrooms of the genus *Lactarius*. The sale of three different species of the section *Deliciosi* was found, which are also the most frequent in other localities in the west of the country: *L*. *deliciosus s*.*l*., *L*. *indigo*, and *L*. aff. *subpurpureus*. The name of *shara*, *xara*, *jolom xar* (head of xara), or *rujolom xar* (the head of the xara) is properly that of *L*. *indigo*, for an association with *Cyanocorax melanocyaneus*, a medium-sized bird with striking blue feathers commonly known as *shara* or *xara*, frequent in the central highland forests of Guatemala. *L*. *deliciosus s*.*l*. and *L*. aff. *subpurpureus* are also known as *xaras* or *xaritas*, although their color is very different because the first one is orange and the second is pinkish. The generic *xara* or *jarita* is also applied in San Martín Jilotepeque [32], while it changes to *k*’*aquix* in Patzún and Tecpán [30], *tolor* in Comalapa [31], and *amacaria* in Sumpango [40], places where the predominant local language is Kaqchikel. It was striking that some people from the Cruz Blanca village called *L*. *deliciosus tzum cabro* (goat udder) (Table 7). When asked about this name, women and men indicated that it was due to the resemblance to the nipple of a goat. It should be mentioned that *L*. *deliciosus* is actually a complex of species in Guatemala [45, 46], and probably a mix of species are offered for sale in most cases. In San Juan Sacatepéquez, at least two species were observed, which are differentiated by subtle morphological characters. At the microscopic level there are few differences, as Nuytinck et al. [47] have pointed out for this section, so recent phylogenetic methods are being used to correctly identify the Guatemalan and Central American species. *Lactarius* aff. *subpurpureus* is a species that has gone so far unnoticed and that can be confused with *L*. *deliciosus*. The fruiting bodies are usually a little smaller than *L*. *deliciosus* and only rarely were found for sale as separate species, in small baskets, while they are usually offered mixed with *L*. *deliciosus*.

The *anacates*, *K*’*axul*, or *Q*’*axul*, *C*. *cibarius* and *C*. *lateritius*, are the mushrooms that have the highest sales in volume in the market from the beginning until the end of the mushroom season. In purchases of fungi, specimens similar to *C*. *roseocanus* (Redhead, Norvell & Danell) Redhead, Norvell & Moncalvo, a species known from North America, were also acquired. Finally, specimens that were classified as *C*. *confluens* were also collected in the field, so that the *C*. *cibarius* complex in San Juan might include at least four-five species (Tables 2 and 3). It is striking that no sale of specimens of the genus *Craterellus* was recorded, despite being present in the local woods. To date, the only Guatemalan town where *Craterellus* (*C*. *ignicolor* (R.H. Petersen) Dahlman, Danell & Spatafora) is known to be sold is Tecpán [30], although there are several species, all edible, in the country [48].

Another rich group with the same common name is *Ramaria* (*Rujolon äk*, deer horns) that contains *R*. *botrytis*, *R*. *araiospora*, and yellowish *Ramaria* aff. *flava*. Due to the lack of adequate taxonomic keys for Guatemala, specimens acquired in the market were only identified up to genus level, with the exception of the two mentioned above, which are the best known. In this study, there were few specimens observed for sale and in the field. However, *Ramaria* is a genus that produces large, colorful and relatively abundant carpophores in mixed pine-oak forests of the central highlands of Guatemala and is sold more often in other locations such as Comalapa and San Martín Jilotepeque, according to recent observations (Flores, unpublished data).

During this study, we attempted to document traditional knowledge (associations and beliefs) about mushrooms shared among vendors, collectors, and consumers of the municipality. Some beliefs and relationship of the name of fungi with plants and animals emerged thanks to this investigation. Both vendors and collectors mentioned that “*los hongos salen después de los zompopos de Mayo*”, the fungi come out after the *zompopos* of May, which means that the fruiting bodies of the fungi—particularly the edible ones—appear after the appearance of the big ants of the genus *Atta* (*A*. *cephalotes*), better known as *zompopos de mayo* (see Fig. 2). We were also repeatedly told that *anacates* “*crecen en donde pasa la gallina ciega*”, grow where the blind chicken passes, meaning that *Cantharellus* grow in places where larvae of *Phyllophaga* spp. (Scarabidae; *gallina ciega*) are nested, whose mature stage is known as May beetles (*ronrón de mayo*). In some of our field samplings, many “*gallinas ciegas*” were found under oak trees. When visiting a sacred syncretic site on a hill, an old man told us that people bring flowers, sweet breads, cigars, candles, and even mushrooms as offerings. This fact reminds the offering of “mushrooms caps and *pericón*”—*Tagetes lucida*, an aromatic and medicinal herb—to the idols, mentioned in the Popol Vuh. This is the only use of mushrooms as offering reported so far in Guatemala. Since San Juan Sacatepéquez is still mostly inhabited by a native population with ancestral customs and traditions, it is striking not to have found more legends, stories, and beliefs that make reference directly to the Mayan worldview. However, there may be some traditional beliefs linked to mushrooms that have not been shared with the investigators, perhaps due to the lack of trust and confidence in people perceived as foreigners (ethnic differences).

Finally, to expand the ethnomycological information on the municipality of San Juan Sacatepéquez, a survey was conducted among 14 market vendors and 28 workers of Cementos Progreso in Chivoc (see Tables 8 and 9). The results of the survey show notable differences in some questions, depending on whether they were vendors/collectors or workers. Several interesting issues emerged thanks to the survey, about the ecological role attributed to mushrooms, on the intergenerational passage of mycological knowledge, and on selling dynamics. When asked about what mushrooms are, around 50% of the sellers/collectors and workers categorized them as fungi, that is, as entities other than plants or animals; 29% of vendors/collectors considered them as vegetables, and 21% did not know or did not respond. Intriguingly, 7% of cement plant workers identified mushrooms as animals, a category that was not considered by any vendor/collector. When asked about a relation between mushrooms and trees, all vendors responded affirmatively and gave reasons as “the trees feed the mushrooms,” “the trees collect the serene of the night and keep moisture in the roots so that the fungi grow,” “the trees provide shade, fertilizer and roots to grow mushrooms,” “the leaves serve as fertilizer and therefore mushrooms grow,” and “only where there are forests mushrooms grow”. A good 93% of the cement plant workers also affirmed that there is a relationship between trees and fungi. The vast majority of interviewed people (86% of salespeople and 89% of cement plant workers, respectively) acknowledged that the production of edible fungi in the San Juan Sacatepéquez area has declined in the last decade or so. Indeed, the extension of forested areas within the municipality has decreased to expand agricultural and floriculture crops, and for the construction of private and commercial buildings.

**Table 8:** General knowledge about mushrooms in the San Juan Sacatepéquez area according to the ethnomycological survey

Issue	Vendors		Cementos Progreso workers	
	*N*	%°	*N*	%°
Identity of mushrooms	14	100	28	100
Vegetal	4	29	12	43
Fungal	7	50	12	43
Fruity	0	0	0	0
Animal	0	0	2	7
Do not know/no opinion	3	21	2	7
Relationship of mushrooms with trees	14	100	28	100
Yes	14	100	26	93
No	0	0	2	7
Mushroom production according to forest type	14	100	28	100
Oak forest	5	35	9	32
Pine forest	4	29	4	14
Mixed forest	4	29	13	47
Do not know/no opinion	1	7	2	7
Substrate where more mushrooms are produced	14	100	28	100
Forest floor	12	86	27	96
Wood	1	7	0	0
Cow dung	0	0	0	0
Do not know/no opinion	1	7	1	4
Where mushrooms grow	14	100	28	100
Each year in the same place	13	93	21	75
Each year in a different place	0	0	4	14
Do not know/no opinion	1	7	3	11
Same mushroom production 10 years ago	14	100	28	100
No	12	86	25	89
Yes	1	7	2	7
Do not know/no opinion	1	7	1	4
Season of growth of the *Hongo de San Juan*	14	100	28	100
Just in June	4	29	16	56
In winter	0	0	4	14
In other periods of the year	5	35	3	11
June and September	2	14	2	7
From June to October	0	0	1	4
Rainy season no matter the month	0	0	1	4
Do not know/no opinion	3	22	1	4
Knowledge about medicinal mushrooms	14	100	28	100
No	14	100	27	96
Do not know/no opinion	0	0	1	4
Knowledge about the existence of hallucinogenic mushrooms in San Juan Sacatepéquez	14	100	28	100
No	14	100	24	86
Do not know/no opinion	0	0	4	14
Use of hallucinogenic mushrooms in San Juan Sacatepéquez	14	100	28	100
No	14	100	26	93
Do not know/no opinion	0	0	2	7
Relationship between God and mushrooms	14	100	28	100
Yes	12	86	23	82
No	1	7	5	18
Do not know/no opinion	1	7	0	0
Mushroom knowledge transmitters	14	100	28	100
Grandparents	4	29	13	47
Mother	5	35	7	24
Father	1	7	4	14
Friends	1	7	2	7
Cousins	0	0	1	4
Do not know/no opinion	3	22	1	4
Knowledge in relation to:	14	100	28	100
Stories about mushrooms	14	100	4	14
Mushrooms with names of animals	5	35	4	14

Source: information obtained through oral (vendors) and written (cement plant workers) interviews, conducted in the period 2012–2015. See “Materials and methods” for further details

°Approximate

**Table 9:** Commerce of mushrooms in San Juan Sacatepéquez

Issue	Vendors		Cementos Progreso workers	
	*N*	%°	*N*	%°
Use of mushrooms	14	100	28	100
Food	14	100	23	82
Do not know/no opinion	0	0	5	18
Recipients of mushrooms	14	100	28	100
Several people in the market	5	35	13	47
Mushroom resale	5	35	9	32
Gatherers near the village	4	30	0	0
Does not sells mushrooms	–	–	6	21
How to differentiate between edible mushrooms from inedible ones	14	100	28	100
Color	5	37	17	59
Cap with or without scales or warts	2	14	4	14
Stem	2	14	1	4
Ring	2	14	0	0
Texture	0	0	3	11
Gills	0	0	1	4
Volva	1	7	1	4
Taste	1	7	0	0
Cuticle	0	0	0	0
Do not know/no opinion	1	7	1	4
Reasons to cut the foot to the mushrooms sold in the market	14	100	28	100
Cleaning	8	58	18	64
Interferes with the taste	3	21	1	4
None of the above	2	14	3	11
Leave “seeds” to grow new mushrooms	1	7	6	21
Population that consumes more mushrooms	14	100	28	100
Indigenous and ladinos	11	79	14	50
Indigenous	1	7	9	32
Ladinos	0	0	4	14
Do not know/no opinion	2	14	1	4
What you like most about mushrooms	14	100	28	100
Taste	11	79	23	82
Taste/texture	1	7	1	4
Appearance	0	0	0	0
Do not know/no opinion	2	14	4	14
Difference between the *Hongo de San Juan* and the *Hongo de San Pedro*	14	100	28	100
Color	8	58	11	39
Taste	3	21	10	35
Price	0	0	3	11
Place of growth	1	7	2	7
None of the above	2	14	1	4
Do not know/no opinion	0	0	1	4

Source: information obtained through oral (vendors) and written (cement plant workers) interviews, conducted in the period 2012–2015. See “Materials and methods” for further details

°Approximate

As for the transmission of mycological knowledge, it emerged clearly that families are the central hub of this delicate process. Collecting mushrooms is a local tradition that is transmitted within the family nucleus, and only rarely outside. Generally, grandparents and the mother are the ones who teach to look for mushrooms and to differentiate edible from inedible ones. Some of the interviewed people commented about the importance of family traditions with sentences as “grandparents are wiser and have more experience” or “they have more chance to find mushrooms to eat or for sale”. Women vendors stated that mothers can transmit the knowledge because they spend the most time with their children, who accompany them during field activities such as collecting firewood or taking care of livestock. As such, the search for mushrooms becomes a recreational activity and helps strengthening family ties, some commented. The father can also participate in this transmission, although at a lower level: he is usually away from home all day, although it does bring mushrooms home if he works near a wooded area or if he can collect some on the way home. These aspects coincide with what is reported by Montoya and colleagues for Tlaxcala, Mexico: “Mushroom gathering is a social activity among women and children, while men generally like to pick alone,” [49]. Some interviewees (7%) also indicated that one can learn how to collect mushrooms among friends, being these either neighbors or study companions; a minority (4% of cement plant workers) said that this activity can also occur between cousins, the elder teaching the youngest.

According to a significant number of interviewees (37% of collectors/vendors and 59% of cement plant workers) one of the main characters to differentiate an edible mushroom from an inedible one is the color. Also, if the mushroom becomes “purple” when touching it, thus if it stains when bruised, it is not edible. A group of women gatherers said that if a mushroom has “*calzoncito*” or “*pantaloncito*” (panties or pants), i.e. a ring, it is edible and if it does not present it, it should not be consumed. This character must refer to *Amanita*, because it is the most conspicuous ring genus in the area: although there are *Agaricus* and *Lepiota* in the field. During the 5 years of research, we could see only one sell of *Agaricus* sp., by a novel-seller mother with her two children. The mushrooms were bought by another local old woman. The 14% of the vendors indicated as important the presence or absence of “scales” in the cap (pileus) of the fungus, explaining that if there are “scales” (remnants of veil), it is not eaten. A good 7% of the vendors believe that they can distinguish mushrooms according to the taste: if the fungus has a bitter or spicy taste, it is not edible. The respondents also indicated that when they get to try a mushroom, they never swallow it. Some 11% of the workers indicated that if the mushroom’s pileus is sticky to the touch it is inedible. However, many fungi present this characteristic with conditions of high humidity or rain, particularly those of the genus *Suillus*, which nevertheless have appeared in the market in recent years. Many interviewees mentioned that these criteria of identification of an edible mushroom are applied together, to avoid confusions and unfortunate consequences such as poisoning. It is striking that no one could remember any cases of intoxication due to mushroom consumption in San Juan Sacatepéquez.

Question about selling/purchasing dynamics also revealed interesting social aspects. When asked “to whom do you sell the mushrooms?”, 35% of the vendors indicated that the main part goes to market people, 35% to resellers, and 30% to other collectors in the village, which indicates an active trade in sale and resale of mushrooms. In the case of cement plant workers, 47% responded that mushrooms collected are mainly destined for sale in the market, 32% resale them and only 21% do not sell and eat them. Although different in percentages, these data coincide in the fact that mushrooms are mainly destined for sale, both for collectors of the municipal villages and cement workers and/or their families. As for the characteristics of mushroom consumers, 79% of vendors responded that both Kaqchikel people and ladinos purchase and consume mushrooms, making no clear distinction in this attitude among these groups. On the contrary, cement plant workers remarked that Kaqchikeles are those who consume more mushrooms with respect to ladinos. One of the collectors mentioned that Kaqchikel women, housewives of San Juan Sacatepéquez, are the ones who buy mushrooms the most because they have no chance of getting them in the field and/or because their family is engaged in non-agricultural occupations.

### Mycodiversity in Guatemala: research perspectives

Although mycological research has been going on in Guatemala since more than a century now [50–52], and despite recent efforts by an increasing number of mycologists, both local and international, knowledge on the mycobiota of Guatemala is still very poor. The last comprehensive checklist reports some 350 species of macromycetes (31 ascomycetes and 319 basidiomycetes) occurring in 163 genera and 20 orders [15]. Since then, fresh studies have focused on the full morpho-anatomical and molecular characterization of the ectomycorrhizae formed by *Lactarius rimosellus* on *Quercus* [53], on solving complexes of cryptic species in several genera, including *Lactarius* and *Boletus* [45, 54, 55], on the diversity and fruiting dynamics of *Marasmius* in the Lachuá Ecoregion, the largest remaining tropical forests in Guatemala [56], and on describing the ectomycorrhizal fungi collected in natural stands of *Pinus caribaea* in the Petén lowlands [57]. Also, new species of local ascomycetes were identified [58], and the analysis and description of new species of anamorphic fungi carried out [59]. With the data originated from molecular and phylogenetic studies, the number of genera has increased, especially in Boletales, and new species were discovered in the eastern zone of the country (Flores and Simonini, in preparation). Needless to say, much remains to be done for such a megadiverse country. The ectomycorrhizal landscape of Guatemala, for example, is still severely underexplored, compared to what is known globally [60, 61]. In this context, research is underway on the diversity of key ectomycorrhizal genera, such as *Lactarius* and *Amanita*. Virtually nothing is known about hypogeous mushrooms; “No native truffles have yet been documented, but they undoubtedly exist,” recently commented David Pilz and colleagues on this issue [62]. Entire areas of the country—such as Sierra de Las Minas, a mountain range that runs through the country’s central-eastern highlands and one of the most important biosphere reserves in America [http://www.unesco.org/mabdb/br/brdir/directory/biores.asp?code=GUA+02&mode=all]—are virtually unexplored from the mycological point of view. Finally, ethnomycological investigation is far to be complete, as the present study has shown, with the significant diversity of edible species (including undescribed ones) found in San Juan Sacatepéquez (see also https://mayansandmushrooms.wordpress.com/about/). Research should be directed not only to recording the consumption of edible mushroom species, but also to disclosing the traditional use of medicinal and hallucinogenic mushrooms [63].

## Conclusions

What we have unveiled thanks to our study is the contemporary wealth of Kaqchikel culture for what concerns mushrooms, demonstrating that mushrooms continue to be culturally and economically important for these communities despite the erosion of traditional knowledge (see also [64]). However, the roots of this traditional knowledge reach deep in history, well into pre-Columbian times. Mushroom stones, as mentioned above, are a clear indication of this. But also, other hints suggest that the Kaqchikel area shared with other Mesoamerican people the use of mushrooms for purposes other than simply food. “That the highland Maya knew the inebriating mushrooms is proven by a number of Mayan word lists for the Cakchiquel linguistic area around Guatemala City and Antigua. The lists that I have seen are mostly handwritten and experts date them from the end of the seventeenth century, though they bear no date.” This is how R. Gordon Wasson discusses the mycolatric practices that possibly took place in the Guatemalan highlands [65], with the officiants under the inebriating effects of *Psilocybe* and maybe other mushrooms [66], as the terms *hongo que emborracha*, ‘mushrooms that inebriates’, and *otros* [*hongos*] *que embriagan*, ‘others [mushrooms] that inebriate’, present in those ancient Kaqchikel word catalogs seem to indicate [65]. “The Maya highlands and the highlands of what is now Mexico are essential areas of Mesoamerica, where there was an active give-and-take in warfare and trade throughout history and prehistory. Here is for me conclusive evidence that the use of entheogenic mushrooms existed in the highlands of what is now Guatemala. An anthropological trait as important as the use of entheogens would inevitably characterize the whole of the cultural entity known as Mesoamerica,” Wasson remarked [65].

With respect to our initial hypotheses, the expectations as for a significant diversity in the number and types of mushrooms offered for sale was completely fulfilled, confirming the strong mycophily of the people inhabiting this sector of the Guatemalan highlands. Although the consumption of wild mushrooms seems to be highly polarized, with a few species that are highly appreciated and intensely traded (e.g., *A*. *caesarea* complex, *anacates*, *Hypomyces lactifluorum*, *pancitas*), the composition of the panel of species offered for sale is apparently dynamic, in the sense that new ones (e.g., *Gastropila*, *Cortinarius* spp., *Tylopilus*) are added, depending on availability at collection sites. This suggests that the criteria used to distinguish edible from inedible (or even toxic, like might well happen with *Cortinarius*) mushrooms are applied in a flexible manner, and fresh ‘knowledge’ is stratified over traditional one, probably after successful attempts. The ways and manners of this process deserve further attention.

The significance of macrofungi conservation, in virtue of their ecological role and their cultural and socio-economic importance, is increasingly appreciated. Although there is still a long way to go before these organisms receive the attention and protection they deserve, macrofungi are starting to be considered in several countries, in North America, Europe, and elsewhere, and plans to protect and manage their diversity drafted (e.g., [67, 68]). Guatemala is certainly a place where all these considerations and attentions are in their very infancy, but our investigation on Kaqchikel ethnomycology offers the opportunity to make some relevant recommendations. For example, we believe that it could be useful to create educational programs for the population to indicate the importance of fungi in the forest and nature, particularly for the conservation of the soil and the forests themselves, as well as educating on the importance of not picking immature mushrooms, so to favor sporulation and avoid the decline of economically important species such as *Amanita*, *Boletus*, and *Cantharellus* (especially the latter, as we discussed above, is particularly in high demand). The fact that both vendors and collectors did recognize that the abundance of wild mushrooms declined considerably in the last decade (Table 8), generally indicating the mutated rain regime as the possible cause, testifies that lay people are aware of the delicate balance governing mushroom sprouting and growth, indirectly pointing to climate change as the reason. On the other hand, intensive gathering of mushrooms as a consequence of increasing demand might have serious consequences. While it is not straightforward to link increased collection amounts of wild mushrooms with their decline in natural settings, habitat destruction due to excessive human pressure on forests will certainly be detrimental, both for the ecosystem and for the income of many families that count on mushroom picking as an important economic resource. Again, an equilibrium is necessary. Therefore, it would also be crucial to promote reforestation campaigns with local pine and oak species, as well as avoiding intensive logging in forest remnants that act as the source of local fungal germplasm, also teaching about the advantages of sustainable forest management and their fungal productivity. Last, but not least, more ethnomycological research in Guatemala is necessary to avoid the loss of ancestral knowledge and preserve the cultural richness of the Mayan peoples in the face of the strong economic and social pressure that drives rapid and irreversible changes in lifestyle.

## Additional file

Additional file 1: **Figure S1.** A map of the forested areas of the Department of Guatemala and the Municipality of San Juan Sacatepéquez (top left corner of the map). Source: SIFGUA (http://www.sifgua.org.gt/Index.aspx). Reproduced with permission. (PDF 6900 kb)

## Data Availability

All the data obtained and materials analyzed in this research are available with the corresponding author and R.F.A.

## Footnotes

1 For authors of fungal names, where not specified, see Tables 2 and 3
