# Supplementary material for: Ethnomycological knowledge among Kaqchikel, indigenous Maya people of Guatemalan Highlands
Source: J Ethnobiol Ethnomed. 2019 Jul 17;15:36. doi: 10.1186/s13002-019-0310-7 (PMC6637636; doi:10.1186/s13002-019-0310-7)
Supplement: Supplementary file 1 — Figure S1. A map of the forested areas of the Department of Guatemala and the Municipality of San Juan Sacatepéquez (top left corner of the map). Source: SIFGUA (http://www.sifgua.org.gt/Index.aspx). Reproduced with permission. (PDF 6900 kb) [file 13002_2019_310_MOESM1_ESM.pdf]

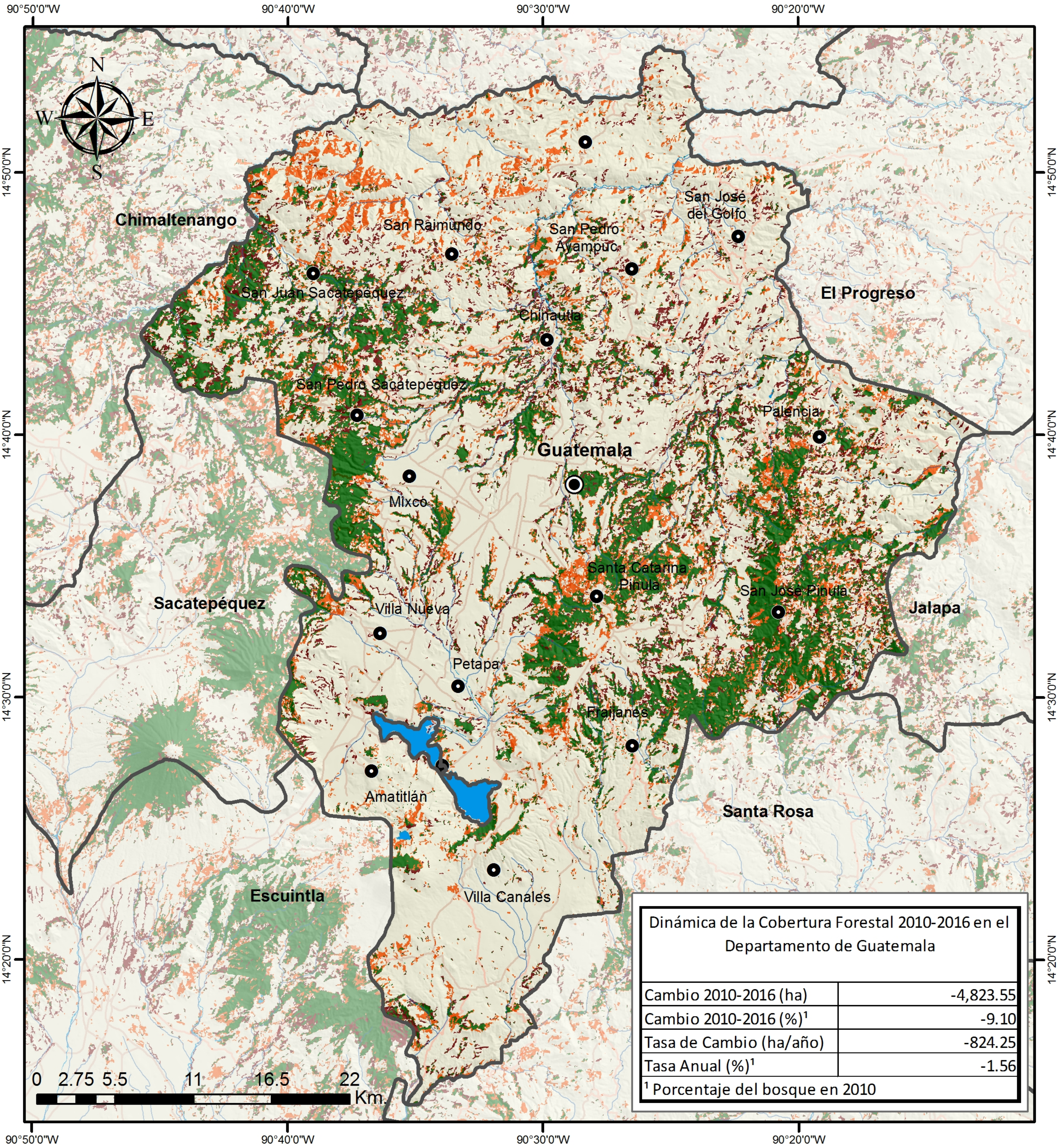

Dinámica de la Cobertura Forestal 2010-2016 en el Departamento de Guatemala

Cambio 2010-2016 (ha)	-4,823.55
Cambio 2010-2016 (%)[1]	-9.10
Tasa de Cambio (ha/año)	-824.25
Tasa Anual (%)[1]	-1.56

[1] Porcentaje del bosque en 2010

Leyenda

- Límite departamental*
- Cabecera departamental
- Cabecera municipal
- Asfaltado
- No asfaltado
- Ríos principales

*Los límites no son autoritativos

Dinámica de la cobetura forestal 2010-2016

- Bosque
- Sin Bosque
- Ganancia de bosque
- Pérdida de bosque
- Cuerpos de agua

Dinámica de la cobertura forestal 2010 - 2016 en Guatemala

Elaborado por:
Instituto Nacional de Bosque -INAB-
Consejo Nacional de Áreas Protegidas -CONAP-
Ministerio de Agrícultura, Ganadería y Alimentación -MAGA-
Miniterio de Ambiente y Recursos Naturales -MARN-
Universidad del Valle de Guatemala -UVG-
Universidad Rafael Landivar -URL-

Escala del estudio 1: 50,000
Sistema de Coordenada Geográfica WGS 1984
Fuente adicional
Guatemala, Abril de 2019
